# Helpful factors of group cognitive behavioral therapy in overweight and obese college students

**DOI:** 10.3389/fpsyg.2025.1585765

**Published:** 2025-09-12

**Authors:** Zixian Zhao, Yuting Zhang, Dan Zhao

**Affiliations:** ^1^Student Mental Health Education and Consultation Center, Chongqing Normal University, Chongqing, China; ^2^School of Education Science, Chongqing Normal University, Chongqing, China

**Keywords:** group cognitive behavioral therapy, helpful factors, thematic analysis method, overweight and obese college students, qualitative research

## Abstract

**Objective:**

To explore the helpful factors of group cognitive behavioral therapy in overweight and obese college students.

**Methods:**

Twelve overweight and obese college students were selected to receive group cognitive behavioral therapy, and semi-structured interviews were conducted to gather insights on their perceived helpful factors during the intervention, and thematic analysis was employed to process the interview data.

**Results:**

The cognitive-behavioral weight loss group intervention had three categories of helpful factors: (1) “Cognitive Adjustment and Skill Learning,” including thought recognition and transformation, emotional experience and acceptance, motor learning and practice, dietary adjustments and changes, and knowledge assimilation and experimentation. (2) “Group process and motivational factors,” including a sense of belonging and cohesion, information transfer and facilitation, and a sense of self-efficacy. (3) “Gains and overall results,” including healthy living and weight loss, renewed hope, and a positive mindset.

**Conclusion:**

Group cognitive behavioral therapy achieves group weight loss through the synergistic effects of multiple factors.

## 1 Introduction

Overweight and obesity are characterized by the abnormal or excessive accumulation of body fat, which can adversely affect health. These conditions are typically classified by the Body Mass Index (BMI). Numerous studies have highlighted a high prevalence of overweight and obesity among college students (Zhang et al., 2020; You, 2021; Han and Wang, 2021). The 8th National Survey on Students’ Physical Fitness and Health further underscores this trend, indicating a rising proportion of students affected by these conditions (Department of Physical Health and Arts Education, Ministry of Education, 2021). Research has consistently shown that overweight and obese college students face various physical and mental health challenges. Excess weight increases bodily strain, leading to slower movement, reduced cardiorespiratory fitness, and heightened risks of diabetes, heart disease, and cancers such as those of the uterus, thyroid, and mammary glands. Additionally, it negatively impacts overall physical fitness (Chen et al., 2024; Zou, 2023; Dal Grande et al., 2009; Chen et al., 2022; Li and Chen, 2022; Lee et al., 2019). Li and Wang (2015) found that overweight and obese college students exhibit lower self-evaluation and self-confidence, along with heightened interpersonal sensitivity compared to their normal-weight peers (Ma, 2016). Shang et al. (2021) also noted that these conditions can lead to depression, anxiety, and other negative emotional states among college students. Cognitive Behavioral Therapy (CBT), developed by A. T. Beck in the 1960s, is a systematic, goal-oriented, short-term psychotherapeutic approach designed to address depression, anxiety, and other irrational cognition (Beck, 1979). CBT aims to modify individuals’ cognitive, emotional, and behavioral patterns to resolve psychological issues. Group cognitive behavioral therapy (GCBT) extends the principles of CBT to a group setting, enhancing treatment efficiency and cost-effectiveness. GCBT not only serves more individuals but also reduces time and financial costs compared to individual therapies (Okumura and Ichikura, 2014). GCBT has demonstrated significant efficacy in treating a range of clinical psychological issues, including social anxiety, depression, bulimia nervosa, and obsessive-compulsive disorder (OCD) (Bernik et al., 2018; Morrison, 2001; Oei and Dingle, 2008; Lloyd et al., 2015; Qiu et al., 2013; Chen et al., 2003). In some cases, GCBT has outperformed individual cognitive behavioral therapy (ICBT). GCBT, incorporating health education, physical exercise, and modern information technology, has shown substantial improvements in dietary behaviors, psychological functioning, and weight management among severely obese individuals, particularly those preparing for bariatric surgery, obese children, and the general obese population (Cynthia et al., 2020; Moraes et al., 2021; Liu et al., 2023; Teeriniemi et al., 2018).

Following the success of interventions, researchers often investigate their underlying mechanisms. Understanding these mechanisms allows for more targeted intervention strategies and enhances their effectiveness by identifying crucial components and significant transformation phases. Recent studies suggest that cognitive behavioral changes contribute to weight reduction. However, the specific mechanisms through which cognitive behavioral therapy aids in reducing overweight and obesity remain unclear, with most findings based on limited quantitative evaluation criteria. The effectiveness of GCBT in addressing overweight and obesity among college students is still under investigation. This limitation restricts the diversity of interventions and fails to fully meet the mental health and weight management needs of college students. This study aims to analyze and validate the application of GCBT for overweight and obese college students. By conducting qualitative research, it seeks to explore the group process, members’ progress, and feedback to support and enhance the use of GCBT in treating overweight and obesity among college students.

Given the above context, the present study plans to conduct semi-structured interviews with overweight and obese university students undergoing group cognitive behavioral therapy. The objective is to explore the various helpful factors they experience during group activities, thereby providing a scientific basis for optimizing the program and improving the intervention’s effectiveness.

## 2 Materials and methods

### 2.1 Participants

This study involved university students with a BMI ≥24 kg/m^2^, aged over 18, who voluntarily participated in a weight loss group therapy after signing confidentiality and consent forms. Exclusions included mental health disorders, physical illnesses preventing exercise, or current obesity treatments. A total of 24 university students participated in this group. They were randomly assigned to the experimental and control groups, with 12 in the experimental group (2 males and 10 females) and 12 in the control group (3 males and 9 females) without intervention. Both the experimental and control groups completed all pre-test and post-test assessments.

Using purposive sampling, target subjects were selected from cognitive behavioral therapy participants based on two criteria: significant weight loss and completion of psychometric measures, and participation in at least five group activities. Twelve members were invited for interviews, with 11 agreeing and one declining due to timing issues. Notably, the majority of interview participants were female (10 out of 11), which may reflect a gender-specific perspective on the perceived helpful factors of the intervention. Details are in Table 1.

**Table 1:** Basic information of study participants.

Respondent code	Gender	Pre-intervention weight (kg)	Post-intervention weight (kg)	Group counseling participation
E1	Women	64.1	59.4	5
E2	Women	61.0	55.5	8
E3	Women	68.6	65.3	5
E4	Women	66.8	66.3	5
E5	Women	81.8	74.3	8
E6	Women	92.5	91.1	7
E7	Women	74.1	68.9	7
E8	Male	95.1	91.1	7
E9	Women	60.5	59.8	7
E10	Women	76.5	72.0	5
E11	Male	77.2	72.4	8

### 2.2 Procedures

#### 2.2.1 Group cognitive behavioral therapy

A self-developed group cognitive behavioral therapy program for university students’ weight reduction, based on CBT intervention theory, included eight units covering group formation, problem-solving, emotion recognition, self-confidence training, and termination. Sessions lasted 1.5 h weekly for 8 weeks (for specific content, see Table 2). The lead facilitator was a certified counselor from Chongqing Normal University with extensive training and experience in CBT and group counseling, and was supported by two postgraduate co-leaders receiving regular supervision.

**Table 2:** List of group programmes.

Sessions	Objectives	Element	Homework
First time we meeting and getting to know each other	Relationship building and creating a climate of security and trust	The leader and co-leader introduce themselves and explain the group support setting and group process to the members; members briefly describe their expectations of group counselling; members discuss the rules of the group process; create benefit response cards.	Read the benefit response card and record your diet every day
Second session: exploring directions	Sharing knowledge about weight reduction. Establishment of specific objectives.	Review homework; members each share what they know about weight reduction; discuss how to eat healthier/exercise significantly at school; complete the decision making worksheet, setting goals worksheet.	Group up at the end of the group, in pairs and threes, to monitor and supervise each other
Third session: problem-solving	Address the factors that interfere with achieving your goals and learn to eat in a positive way.	Discuss progress towards goals and obstacles and challenges, how to cope with stimuli in the process of goal achievement; discuss how to address factors that interfere with goal achievement; eat biscuits, sultanas, etc. in a positive way and share feelings.	Experience eating slowly with awareness and write down the parts of the body that are used during the eating process
Fourth Emotional Management	Assist members in identifying the relationship between their emotions and eating.	Feel your body in a positive way, assessing hunger and distinguishing between hunger, hankering and appetite; eat your dinner on top of the positive thought eating cookie and experience satisfaction and fullness; discusses the nine primary emotions and how they relate to emotional eating and cravings.	Record scenarios and reasons for choosing to eat when you are full again
Fifth session: cognitive adjustment	Learn to recognize destructive automatic thoughts and make adjustments.	Members discuss progress towards goals, obstacles and challenges; discuss with members their own destructive ideas and thinking errors in weight reduction; explore how to replace negative thoughts with more neutral/positive thoughts or positive attitudes.	Schedule exercise daily and record it.
Sixth session: stimulus control	Discuss the role of stimulus control in changing lifestyles.	Discuss with members strategies for coping with the challenges posed by lifestyle changes by controlling stimuli from dietary styles, exercise and related habit changes.	As before
Seventh session: self-confidence training	Discuss progress towards goals, obstacles and challenges; learn skills and methods for coping.	Members discuss progress towards goals, obstacles and challenges and the skills and methods available to cope when stuck; Use mindfulness techniques and behavioral techniques to calm yourself.	As before
Eighth session: consolidation and termination	Summarise the takeaways, discuss the hindrances and solutions at the end of the day, and bid farewell to each other as a group.	Summarise the CBT strategies and takeaways learnt; common and specific triggers and warning signs of a possible rebound are discussed, as well as how to manage these in the future; share regrets about being in the group and what else you hope to do in the group; send each other good wishes.	

The objectives of this group cognitive behavioral therapy were: on the one hand, through participating in the group cognitive-behavioral counseling, the subjects can understand the relevant principles of cognitive-behavioral therapy and the related knowledge of weight loss and apply the acquired knowledge and experience to their studies and daily life. On the other hand, to guide members to set reasonable weight loss goals, correct irrational cognitions, promote a positive self-understanding, enhance self-efficacy, and reduce anxiety levels. In addition, the effect of weight reduction is maintained through a series of behavioral training and reinforcement to improve the level of healthy eating and exercise and to improve the lifestyle.

#### 2.2.2 Data collection procedures

This study used interviews to collect data. The researcher adapted Elliott’s (2011) “change interview outline” to fit the study’s context, forming a draft centered on three core elements: (1) changes in intervention members, (2) their perceptions of these changes, and (3) their emotional states. After discussion, a formal semi-structured interview outline was created, covering topics such as: ① feelings and changes from participating in the group, ② memorable events and their impact, ③ changes in diet and exercise, ④ personal efforts and factors contributing to change, ⑤ met or unmet expectations, and ⑥ the most helpful aspects and unique characteristics of the group.

Within a week of the end of the group intervention, the researcher conducted individual face-to-face interviews with 11 participants in the school counseling room. Before the formal interviews began, the researcher again explained to the participants the purpose of the study, the principles of confidentiality and information use, and let the participants know that they had the right to ask questions at any time. After explicit consent was obtained from the participants, the entire interview was audio-recorded.

#### 2.2.3 Data organization and analytical approach

The researcher transcribed and numbered each audio recording verbatim, labeling them based on interview content. Participants were labeled as E, with sentences numbered sequentially (e.g., E1-1, E1-2 for the first interviewee). The transcript totaled 33,945 words and 128 min of recordings, averaging 10 min per interview. All text data were imported into Nvivo12 for qualitative analysis, with Microsoft Excel used for preliminary data organization and cross-checking coding consistency.

Data analysis followed Braun and Clarke’s (2006) thematic analysis method, involving five stages: ① Familiarizing with raw data through transcription and repeated reading; ② Generating initial codes by extracting meaningful units from the text; ③ Identifying themes by organizing and classifying initial codes; ④ Reviewing and refining themes using constant comparison, creating a “theme map”; ⑤ Defining and naming themes by analyzing and examining sub-themes and higher-level themes. Theme saturation was determined when no new codes or themes emerged from three consecutive interviews.

For specific coding, the researcher initially coded all texts. Then, another researcher in the field validated the texts along with three random texts to assess the accuracy of the initial coding. Coding discrepancies (occurring in approximately 15% of codes) were resolved through consensus discussions with a third researcher when necessary. Based on this, themes were extracted from the initial coding through discussion, and more advanced themes were extracted through repeated comparison, merging, and splitting to form a three-level coding framework.

## 3 Results

### 3.1 Thematic coding of helpful factors for weight reduction

After thematic analyses, 25 initial codes were obtained, 10 more themes were extracted, and three higher-level themes were obtained. Respondents indicated two main factors for positive change, namely “Cognitive adjustment and skill learning” and “Group process and motivational factors.” They also included the effect of achieving group goals, “Group gains and overall effects.” (see Table 3).

**Table 3:** Thematic coding of factors contributing to weight loss effects.

Level 3 coding (more advanced topics)	Level 2 coding (subject coding)	Level 1 code (initial code)
Cognitive adjustment and skill learning	Emotional experience and acceptance	Group counseling to reduce anxiety, peace of mind, sense of self-efficacy
	Thought recognition and transformation	Changing old beliefs, rationalizing eating, rationalizing weight, identifying hunger
	Dietary adjustments and changes	Experiencing positive eating, changing eating habits, and learning about diets
	Motor learning and practice	Increased frequency of exercise, knowledge of exercise
	Knowledge assimilation and experimentation	Weight loss knowledge acquisition, weight loss knowledge implementation, social learning and development
Group process and motivational factors	Sense of belonging and cohesion	Relaxed atmosphere, group motivation, like-minded friends
	Information transfer and facilitation	Members share experiences and methods, encourage each other to make progress together
	Sense of self-efficacy	Have more confidence in yourself and recognise your own behavior
Gains and overall results	Healthy living and weight loss	Healthy lifestyle and weight loss
	Renewed hope and a positive mindset	Hopeful and positive mindset towards weight loss

#### 3.1.1 Cognitive adjustment and skill acquisition

This section highlights the improvements and changes in thinking and mood resulting from group counseling, as well as the acquisition and application of weight loss knowledge, primarily focusing on exercise and diet. Most interviewees reported that group cognitive behavioral therapy enhanced their emotional well-being and provided them with new insights into loss. They also noted that they gained substantial knowledge and were able to apply it practically.

Emotional experience and acceptance: Seven respondents indicated that the program helped them accept and manage negative emotions, boosting their self-confidence and reducing anxiety. For instance, one participant stated, “I feel less anxious about weight loss now. The more I learn, the more relaxed I become, which helps me stay calm and lose weight more effectively” (E2), This shift occurred as acquiring knowledge provided a sense of control—understanding the science behind weight management alleviated excessive worry. Another mentioned, “During group counseling, my unpleasant emotions dissipated, replaced by a sense of inclusion and comfort. I feel more self-compatible now.” (E9)This reflects how the group’s supportive environment fostered psychological safety, allowing members to lower their emotional defenses. A third participant added, “Understanding healthy weight loss methods has made me calmer and less anxious” (E4). This demonstrates that by adopting practical behavioral strategies, members developed confidence in addressing weight-related challenges, leading to positive emotional changes.

Thought recognition and transformation: Participants reported gaining new perspectives and developing a more logical approach to weight management. One interviewee explained, “My mindset has changed. Before, I would get anxious if I did not lose weight immediately. Now, I understand that body fat reduction is a gradual process, and this new perspective has helped me stay calm” (E10), showing how recognizing the natural progression of weight loss reduced performance pressure. Another shared, “I realized that just watching my weight will not help. Even if I run in Tennessee, no one will notice. This shift in thinking has made me more rational” (E5), indicating that distancing from external validation helped focus on personal health goals. A third participant noted, “I’ve learned to have a healthier relationship with food, avoiding both resistance and pathological cravings. My view is now more balanced” (E9), demonstrating how the intervention helped establish a moderate approach to eating.

Dietary adjustments and changes: After participating in the cognitive-behavioral weight loss program, all interviewees reported learning about rational eating and making significant changes to their dietary habits. One participant remarked, “I’ve started to savor my food more and no longer eat hastily as I used to” (E10), demonstrating how mindful eating techniques helped develop healthier consumption patterns. Another said, “My diet has improved noticeably. I used to eat a lot of unhealthy, oily, and salty foods. Now, I focus more on nutritional balance and eat significantly less” (E5), showing how nutritional education effectively modified food choices and quantities. A third participant added, “The program’s guidelines on portion control have become a habit for me, and I continue to follow them” (E1), indicating the successful internalization of behavioral strategies into daily routines.

Motor learning and practice: All respondents reported gaining knowledge about exercise and increasing their physical activity levels. One participant shared, “I used to find exercise painful and compulsory, but now I feel motivated and enjoy it more. The initial soreness has diminished, and I find the process more pleasant” (E11), showing how gradual adaptation transformed exercise from a chore to an enjoyable activity. Another mentioned, “I’ve started to incorporate more exercise into my daily routine. Instead of sitting in the car, I walk whenever possible. Walking is exercise, and doing more of it is beneficial” (E5), demonstrating how simple lifestyle changes can effectively increase physical activity. A third participant noted, “I don’t focus intensely on exercise, but I make an effort to walk more and intentionally increase my physical activity” (E9), indicating that sustainable behavior change does not require extreme measures, but rather consistent, manageable adjustments.

Knowledge assimilation and experimentation: Interviewees expressed that they gained valuable knowledge from the group sessions, including insights into diet, exercise, and other weight reduction techniques, which also positively impacted their social interactions. One participant stated, “I’ve learned a lot about the science of weight loss. I apply these principles on my own and find them effective, which motivates me to persist” (E5), showing how scientific understanding translated into practical self-management skills. Another shared, “One key point I learned is that hunger and thirst centers are close in the brain. Sometimes, what feels like hunger might actually be thirst. This understanding has helped me manage my eating habits better” (E2), demonstrating how physiological knowledge led to more mindful consumption patterns. A third participant added, “Whether it’s about relationships, emotional interactions, or weight loss knowledge, I’ve gained a lot. The group support has been very fruitful for me” (E4), highlighting the multidimensional benefits of the group learning experience.

#### 3.1.2 Group processes and motivational factors

This section primarily examines the intrinsic functions of the group itself. Under the guidance of the leader and co-leader, through active cooperation and participation of group members, a relaxing, pleasant, safe, and comfortable atmosphere is created. This environment provides members with positive emotional experiences and opportunities for personal growth.

Sense of belonging and cohesion: All interviewees reported feeling comfortable and cheerful within the group. The members, being like-minded, interacted like friends, creating a natural environment conducive to openness. As one participant noted, “I think the overall atmosphere in this group is more relaxed and pleasant. We discuss various weight loss methods and habits together, which feels very enjoyable” (E10), showing how the supportive group dynamic fostered a positive learning environment. Another shared, “Previously, I was on my weight loss journey alone. Suddenly finding a group where people can share their problems or progress made me feel like I’ve found a place for collective improvement” (E7), illustrating how peer support alleviated the isolation often felt in individual weight loss efforts. A third participant described, “Although we may not seem very familiar with each other, we are mentally connected. It’s like we’re the real-life version of an internet chat room. Despite not being closely acquainted, we can understand each other through mannerisms, share common goals, and feel united in purpose” (E5), demonstrating how shared objectives created strong psychological bonds among members.

Information transfer and facilitation: Interviewees reported that group therapy facilitated weight loss and personal improvement through experience sharing. One participant stated, “There are people here who share similar experiences. They suggest methods they find effective and share healthy approaches, helping me realize my situation is not as severe as I thought, which keeps me positive” (E3), showing how peer modeling normalized challenges and provided practical solutions. Another explained, “By listening to others’ experiences and sharing my own, I gain multiple approaches and substantial support. The mutual encouragement during daily check-ins is particularly valuable” (E2), demonstrating how bidirectional knowledge exchange enriched everyone’s toolkit for change. They added, “When we encourage each other, share methods, and provide strength, it’s a reciprocal process. Both giver and receiver benefit equally,” highlighting the synergistic nature of group learning where teaching reinforces the teacher’s own understanding while helping others.

Sense of self-efficacy: Five members reported that group communication and engagement, combined with their personal efforts to change, enhanced their self-trust, confidence, and ability to approach difficulties rationally. One participant shared, “These discussions have boosted my confidence in myself. Even during the busy finals week, it prevents me from feeling overwhelmed. I feel more in control of my situation, and things are moving in the right direction” (E9), demonstrating how group validation strengthened coping abilities during stressful periods. Another noted, “After losing weight, people generally feel better, whether in social interactions or personal activities. This improvement boosts confidence and ease” (E3), showing how tangible progress reinforced self-assurance across life domains. A third participant reflected, “Realizing that I’m not as overweight as others say, and that my situation is not as severe as described, helps me accurately assess my current status” (E4), indicating how objective self-reappraisal replaced distorted perceptions through group feedback.

#### 3.1.3 Gains and overall results

This section focuses on the fulfillment of group members’ expectations and the achievement of their goals, primarily highlighting the effects and impacts of the weight-loss group counseling on their lives. It also explores the long-term changes and benefits that participants have gained from this group counseling experience.

Healthy living and weight loss: Group participants reported that the counseling sessions significantly aided their weight loss efforts and helped them integrate healthy weight-loss practices into their daily routines, leading to a healthier and more structured lifestyle. For instance, one participant shared, “I’ve become healthier and have instinctively applied the practices from group therapy to my life. After sitting for long periods, I now make a point to get up and move around, which has made me feel better about my overall fitness” (E9), showing how behavioral changes became automatic through consistent practice. Another participant emphasized the importance of sustainability, stating, “I think I should stick to these habits, not just to improve my appearance but also to ensure a healthier future and enhance my quality of life” (E3), demonstrating a shift from aesthetic goals to holistic health awareness. Reflecting on the long-term impact, one member noted, “There are moments in my daily life when I recall the knowledge and methods shared during the group counseling sessions. These memories motivate me to continue moving forward and maintaining a healthier lifestyle” (E1), highlighting how the intervention created lasting cognitive triggers for healthy choices.

Renewed hope and a positive mindset: ten participants expressed that the weight-loss group helped them regain confidence in their ability to lose weight, instilling a belief that they can achieve meaningful change. They reported feeling more optimistic, calm, and self-assured in their approach to weight management. One participant remarked, “My anxiety about weight has decreased significantly because I’ve realized that weight loss is a positive goal, but even if I don’t achieve it, it’s okay. I’ve come to understand that societal perceptions of obesity are not as severe as the internet portrays, which has alleviated much of my stress” (E2), showing how cognitive reframing reduced perfectionist pressure and social anxiety. Another shared, “I feel good about myself and receive positive feedback from both myself and others. My self-perception has shifted—I now believe in my ability to succeed. Instead of doubting myself, I encourage myself by saying, ‘You’re doing well. Keep going, and you’ll achieve even more” (E3), demonstrating the development of self-compassion and intrinsic motivation through progress validation. A third participant reflected on their journey, stating, “As I became more engaged in the process, I noticed that I was losing weight more effectively and gaining confidence. This transformation was a significant turning point for me, and I truly believe I can continue to improve in the future” (E9), illustrating how behavioral engagement created a virtuous cycle of success and self-belief.

### 3.2 Relationship between factors helping weight loss effect

**Figure 1:**
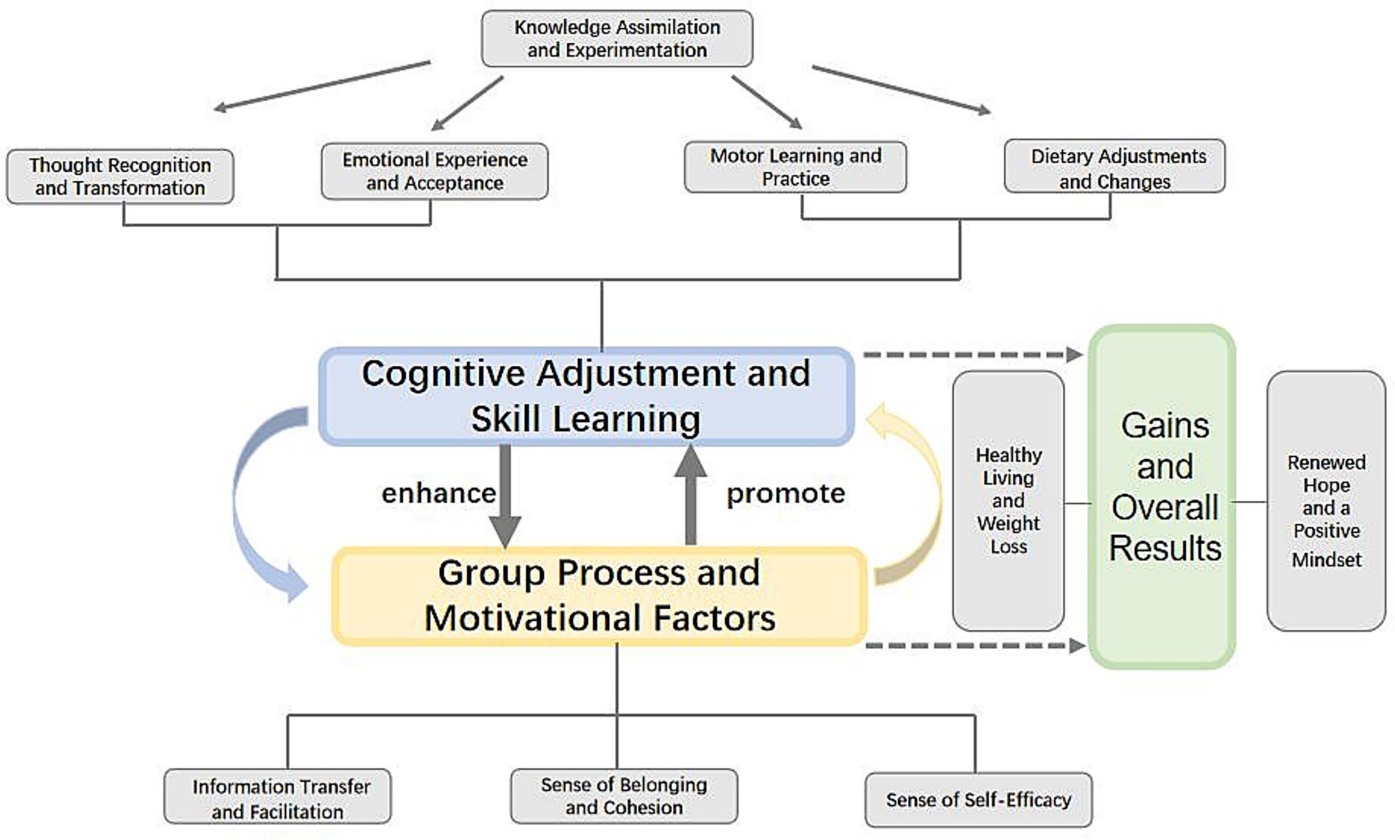
Relationship between helpful factors and group cognitive behavioral therapy.

This study deeply analyses each interviewee’s perceptions of the relationships between the various helpful factors and their attributional logic for their changes. Through individual interviews and cross-case comparisons among the 11 interviewees, the relationships of the helpful factors were finally charted (Figure 1).

#### 3.2.1 The mutual reinforcement between “cognitive adjustment and skill learning” and “group process and motivational factors”

The study highlights the dynamic interplay between “cognitive adjustment and skill learning” and “group process and motivational factors,” which collectively drive the effectiveness of group cognitive behavioral therapy (GCBT) for overweight and obese college students. Cognitive adjustment and skill learning encompassed emotional regulation, thought transformation, dietary changes, and exercise practices, enabling participants to adopt healthier behaviors and rationalize weight-related beliefs. The group process—characterized by cohesion, shared experiences, and mutual encouragement—not only amplified these cognitive-behavioral gains but was simultaneously strengthened by participants’ increasing mastery of therapeutic skills. This reciprocal relationship created a virtuous cycle where cognitive-behavioral improvements enhanced group engagement, while the supportive group environment reinforced continued skill application and development. Yalom’s therapeutic factors and Burlingame’s model of group processes provide a theoretical framework for understanding this synergistic interaction, where individual change and group dynamics become mutually catalytic elements in the therapeutic process.

#### 3.2.2 Holistic impact of the GCBT model

The synergistic model demonstrates how cognitive-behavioral adjustments and group dynamics interact to generate comprehensive outcomes encompassing weight reduction, improved lifestyle patterns, and enhanced psychological resilience. Participants consistently identified this dual mechanism as fundamental to their sustained success—cognitive techniques served as practical instruments for transformation, while the group environment provided essential social reinforcement and emotional support. This interaction is exemplified by the consistent application of nutritional knowledge through group accountability mechanisms, which effectively translated learned skills into enduring behavioral modifications. The qualitative findings substantiate this reciprocal relationship, highlighting group cognitive behavioral therapy’s distinctive ability to simultaneously target individual cognitive processes and leverage collective motivational forces.

## 4 Discussion

### 4.1 Comprehensive analysis of GCBT intervention effects

The current study implemented a behaviorist learning-based group cognitive behavioral therapy (CBT) program that integrated traditional weight-loss lifestyle change strategies—such as physical activity and dietary guidance—with specific cognitive behavioral techniques. This approach aimed to identify and address obesity-related behaviors, including binge eating and sedentary habits, thereby motivating participants to adopt healthier diets and lifestyles. The study investigated the effects of cognitive-behavioral weight-loss groups on overweight and obese college students from the participants’ perspectives, uncovering the key factors contributing to the program’s effectiveness.

The results highlighted two primary factors that played a crucial role in the program’s success: “cognitive adjustment and skill learning” and “group process and motivational factors.” These elements were found to reinforce each other, driving progress collectively. Additionally, the outcomes were encapsulated under two overarching themes: “group gains and overall effects” and “cognitive-behavioral weight loss group dynamics.” The former summarizes the immediate and long-term impacts of the program, while the latter emphasizes the sustained influence of the group-based approach. Together, these findings demonstrate the program’s effectiveness in fostering meaningful behavioral and cognitive changes among participants.

#### 4.1.1 Discussion of the effectiveness of group cognitive behavioral therapy in overweight and obese college students

After an 8-week cognitive behavioral therapy (CBT) intervention, the experimental group achieved an average weight loss of 3.2 kg, representing 4.5% of their total body weight. These results align with findings from previous studies on CBT for overweight and obese individuals (Cooper and Fairburn, 2001). Follow-up assessments indicated significant weight retention, demonstrating the program’s sustained effectiveness. Participants reported that the group met their expectations and effectively supported their weight-loss efforts. Many expressed gratitude for the transformative impact of the program, with some attributing personal changes directly to their participation in the group.

These findings suggest that group-based cognitive behavioral therapy is a promising approach for addressing overweight and obesity among university students, corroborating recent research by Moraes et al. For instance, a 12-week group CBT program for 18 obese patients resulted in an average weight reduction of 3.07 kg. The current study further reinforces the potential of CBT as an effective intervention for weight management in this population.

In recent years, the beauty and fitness industry has experienced significant growth, offering a wide array of slimming technologies and weight-loss methods. However, the effectiveness of these popular techniques and approaches often falls short of expectations. Issues such as minimal weight reduction, lack of visible results, and frequent weight rebound are common. The subtle or unsatisfactory effects of weight loss are not solely attributable to the techniques or methods themselves but are also influenced by a critical factor that is often overlooked: the individual’s active participation and mindset adjustment (Xiao, 2020). Research indicates that achieving significant weight loss requires attention to mental health (Zhang and Hhan, 2022).

The group intervention in this study not only addressed physical weight loss but also enhanced participants’ psychological well-being. Participants reported that the group helped them build self-confidence, practice self-acceptance, and adopt a more optimistic outlook on life and the future. Maintaining a positive mindset is particularly crucial when facing setbacks in weight loss. For instance, if individuals constantly think, “I can’t do it anyway,” they are likely to lose motivation, potentially leading to emotional eating, overeating, and subsequent feelings of regret and self-blame (Shao, 2024). Participants noted that the group helped them accept fluctuations and rebounds in weight loss as part of the process, shifting their perspective from seeking quick fixes to embracing a more sustainable approach.

The cognitive-behavioral weight-loss group demonstrated notable success in fostering these changes. Based on the findings, the author recommends group psychological interventions for overweight and obese college students as an effective strategy to reduce anxiety, address negative emotions, and cultivate healthy habits conducive to weight loss. Such interventions can lead to more sustainable and improved weight-loss outcomes.

#### 4.1.2 Group cognitive behavioral therapy on “cognitive adjustment and skill acquisition” for overweight and obese college students

Cognitive-behavioral weight-loss programs have been shown to significantly enhance university students’ “cognitive adjustment and skill learning.” The current study highlights that the skill-learning mechanism of cognitive behavioral therapy (CBT) relies heavily on the development of cognitive-behavioral skills. All participants reported gaining substantial knowledge about scientific weight loss, which motivated them to adopt more mindful dietary and exercise practices. Group cognitive behavioral therapy has proven effective in improving dietary and activity habits, ultimately leading to healthier weight management (Yin, 2022).

However, societal pressures, particularly among young women, often contribute to unhealthy weight-loss behaviors. According to Bahreynian et al. (2015), young women’s dissatisfaction with their weight stems from an unrealistic desire for an extremely thin, “bony” body. This dissatisfaction has normalized unhealthy weight-loss methods, such as extreme dieting, dietary restriction, irregular eating patterns, and other harmful practices (Field et al., 2008). As Liu (2023) noted in her research, many individuals resort to drastic measures like dieting and purging, which, coupled with obesity-related anxiety, have led to severe physical and mental health issues. Van Strien et al. (1986) further emphasized that unhealthy eating habits are closely tied to poor dietary cognition and are often accompanied by psychological symptoms, such as distorted perceptions of food and compensatory behaviors.

The weight-loss experiences shared within the group revealed that most members had previously struggled with unhealthy practices like extreme dieting and irregular eating. These methods not only resulted in insignificant weight loss or rapid rebound but also negatively impacted their emotional well-being, leading to anxiety, depression, low self-esteem, and heightened sensitivity. The group’s intervention helped participants challenge their irrational beliefs about weight loss and reshape their perceptions of weight and self-image. Participants reported noticeable changes in their weight and adopted healthier dietary practices, such as prioritizing balanced meals, maintaining calorie deficits, and understanding nutritional needs. Following the intervention, restrictive eating behaviors—defined as limiting food intake for weight control—decreased significantly (Herman and Polivy, 1975).

Restrictive eating often disrupts the body’s natural equilibrium, triggering physiological defense mechanisms like increased hunger and reduced metabolic rates. Additionally, disinhibitory factors, such as negative emotions or exposure to high-calorie foods, can undermine self-control, further complicating dietary behaviors. The interplay between these factors often leads to counter-regulation, resulting in overeating. However, through mindful eating practices, participants learned to observe and experience sensory sensations in a non-judgmental and neutral manner. This awareness helped them better manage hunger-driven impulses and reduce the tendency to consume unhealthy snacks. Positive thinking further enhanced body awareness, making it easier for participants to recognize hunger and satiety cues. Improved satiety perception, in turn, helped regulate overeating tendencies.

These findings underscore the potential of cognitive behavioral therapy as an effective intervention for overweight and obese individuals in China. As Zhao et al. (2021) suggested, CBT can address both the psychological and behavioral aspects of weight management, offering a holistic approach to achieving sustainable weight loss and improved mental health.

#### 4.1.3 Discussion of group cognitive behavioral therapy on the “group process and motivational factors” of overweight and obese college students

The “Group Process and Motivational Factors” highlight the group as a natural environment for interpersonal interactions, reflecting Yalom’s proposed therapeutic factors such as group cohesion, universality, and renewed hope (Wong, 2011). The experiences of participants in group cognitive behavioral therapy (CBT) align with Burlingame et al.’s framework for effective group interventions (Burlingame et al., 2013). This approach is grounded in Yalom’s theory of group therapeutic factors and integrates unique insights from research on group therapies for psychiatric conditions.

The model begins with the Formal Theory of Change, focusing on “skill learning and cognitive adjustment.” Through CBT interventions, group members acquire skills and undergo cognitive restructuring, enabling them to transform harmful behaviors and thought patterns. The second component emphasizes the “small group process,” illustrating how group dynamics influence mechanisms of change. This segment evolves into “Group Process and Motivational Factors,” which underscores the role of intra-group interactions and dynamics in fostering member growth. Through dialogue, shared experiences, and mutual support, group members feel valued and empowered to initiate change and overcome challenges. The model also incorporates leader, patient, and structural elements. To better capture the group’s motivational impact, we adapted this component, making the model more realistic and better equipped to explain the positive changes facilitated by the group process.

Within the group, the first and second components of the model reinforce each other, reflecting the complexity and diversity of the therapeutic process. These two key elements enable group members to develop positively, grow, and achieve psychological recovery. This study not only supports Burlingame’s empirical model but also provides new theoretical and practical directions for group cognitive behavioral therapy.

Researchers and therapists have extensively examined the interplay between group dynamics and skill learning in group CBT. Through interviews, this study found that group dynamics and skill learning are mutually reinforcing. In the group’s safe, supportive, and open environment, members respect and encourage one another, fostering a sense of belonging and cohesion. This atmosphere makes them more likely to share experiences, learn collaboratively, and take collective action to achieve their goals. Historically, the focus has been on resource efficiency and productivity in group settings, often at the expense of interpersonal relationships. However, many experts now agree that strategies are effectively taught in groups, and group dynamics can either enhance or hinder the learning objectives of CBT. Therefore, process and technique should work in harmony rather than compete (Seligman et al., 2007).

This study suggests that cognitive-behavioral groups require a combination of facilitators to strengthen the mutually reinforcing relationship between “group process and motivation” and “skill learning and cognitive adjustment.” By enhancing this relationship, the therapeutic outcomes of group CBT can be significantly improved.

### 4.2 Predictors of individual differences in intervention response

The participants in this study were primarily undergraduate students, with a significant majority being female. This demographic distribution may reflect several underlying factors:

First, compared to graduate students, undergraduates face greater appearance-related pressure, particularly due to peer comparisons and heightened body image concerns influenced by social media (Scully et al., 2023; Ma, 2024). Additionally, their more structured academic schedules allow for better adherence to weekly group sessions. In contrast, graduate students often have busier schedules, balancing research responsibilities and academic development (Dong et al., 2022), making time management more challenging. Furthermore, graduate students typically possess more mature cognitive abilities and stronger resource integration skills (Yang et al., 2025), enabling them to independently address appearance-related anxieties through various channels. Second, the predominance of female participants may stem from women’s greater susceptibility to negative body image and appearance-related anxiety. They tend to be more sensitive to the “ideal body” standards promoted by the internet and media (Jackson and Chen, 2011; Fardouly et al., 2015), and societal expectations regarding female appearance are often more stringent (Fardouly and Rapee, 2019). These sociocultural factors make women more attentive to body changes and more inclined to seek group support for related issues. These findings suggest that future interventions should account for the varying needs of different student populations, such as designing more flexible scheduling for graduate students or developing group participation strategies tailored to men.

Analysis revealed significant variability in participants’ responses to the intervention. Members who showed greater improvement typically exhibited the following characteristics: they began with confidence in their ability to change and were more proactive in sharing experiences and raising questions during group sessions. Notably, the two participants with the highest attendance rates achieved particularly significant weight loss, which was closely tied to their sustained high levels of engagement—they not only completed homework assignments on time but also actively shared daily food logs in the group chat, encouraging discussions among other members. In contrast, those with smaller improvements tended to be more passive and had relatively lower attendance rates. These differences suggest that intervention outcomes depend not only on the content of the program but also on the depth and consistency of participants’ involvement (Appiah, 2023). While participants who self-reported greater improvement did indeed achieve larger weight reductions, the more critical factor was their shift toward a more positive mindset (Brouzos et al., 2021)—for example, accepting weight fluctuations and viewing weight loss as a gradual process. This highlights the need for future interventions to incorporate more robust engagement incentives, such as setting phased goals and strengthening peer support, to enhance overall participation and adherence.

### 4.3 Implications for intervention optimization

This study reveals a synergistic mechanism between cognitive adjustment and group processes, which provides important guidance for group intervention practice. From the perspective of the underlying mechanism, the acquisition of cognitive-behavioral skills establishes a common foundation for group interactions, while the group environment facilitates the practical application of cognitive changes through social reinforcement and peer modeling, creating a mutually reinforcing virtuous cycle (Cooke et al., 2024). This mechanism suggests that future group designs should particularly emphasize the organic integration of cognitive training and group dynamics. Regarding intervention duration, although the 8-week model adopted in this study achieved significant effects, participant feedback indicated some attenuation in skill application post-intervention. Drawing from the stages of behavior change theory, we recommend implementing a 12-week moderate-intensity intervention in future studies, with the first 8 weeks focusing on intensive cognitive-behavioral training and the subsequent 4 weeks transitioning to a peer-supported maintenance phase, as this may better facilitate lasting behavioral change. Concerning group composition, while the homogeneous groups in this study enhanced member identification, they may have limited experiential diversity. Based on social learning theory (Kärner and Warwas, 2015), we propose adopting a “goal-homogeneous but background-heterogeneous” formation principle in future interventions. This approach maintains consistent weight-loss goals while appropriately incorporating members from diverse academic backgrounds and grade levels, thereby preserving group cohesion while providing richer cognitive perspectives. Additionally, establishing post-intervention online support platforms may help sustain group effects.

These optimization recommendations not only stem from the empirical findings of this study but also align with recent theoretical advances in group dynamics, offering valuable references for future research. Subsequent studies could further explore sensitivity differences to the synergistic effects among different populations and determine optimal intervention parameters.

### 4.4 Shortcomings and prospects of the study

The study found that group cognitive behavioral therapy (CBT) for overweight and obese college students significantly reduced weight, anxiety, and restrictive eating behaviors, as evidenced by both data and participant feedback. However, after analyzing the intervention’s design and implementation, several areas for improvement were identified.

1. Intervention duration: the intervention in this study was conducted over 2 months to address overweight and obesity among college students. Due to various factors, multiple postponements occurred, extending the overall timeline and potentially impacting weight loss outcomes. In future studies, leaders could adjust the group schedule by increasing the number of sessions to 12 and extending the duration of the intervention. This would allow more time to consolidate and reinforce cognitive and behavioral changes among participants, potentially leading to better results. To ensure the highest quality of weight loss outcomes, leaders should also manage group schedules more effectively to minimize delays and postponements.
2. Timing of retrospective interviews: in this study, retrospective interviews were conducted after the completion of all eight group activities within a relatively short timeframe, which may have been subject to memory bias and limited our ability to fully capture participants’ nuanced experiences or systematically examine negative reactions (e.g., resistance, adverse effects) during the intervention. To capture more accurate and immediate insights into participants’ experiences and changes, future studies should not only conduct interviews promptly after each group session but also extend the interview duration. This enhanced approach would provide a clearer understanding of the members’ progress and the impact of each session.
3. Targeted research on participant characteristics: further research could explore the characteristics of overweight and obese college students who benefit the most from cognitive-behavioral groups. Additionally, identifying individuals who show minimal changes and understanding the factors that hinder their success would be valuable. These lines of research are important for tailoring interventions to maximize effectiveness and address the diverse needs of participants.
4. Gender imbalance in the sample: it should be noted that the study sample was primarily drawn from a teacher-training university with inherent gender distribution characteristics (approximate 3:7 male-to-female ratio). While this sampling approach provides valuable data for understanding intervention effects in this specific student population, it may limit the generalizability of findings to other academic institutions. Future research could expand the sample to include diverse university settings to further validate the broader applicability of the intervention approach.

## Data Availability

The original contributions presented in the study are included in the article/Supplementary material, further inquiries can be directed to the corresponding author.
