# Supplementary material for: Helpful factors of group cognitive behavioral therapy in overweight and obese college students
Source: Front Psychol. 2025 Sep 12;16:1585765. doi: 10.3389/fpsyg.2025.1585765 (PMC12463828; doi:10.3389/fpsyg.2025.1585765)
Supplement: Supplementary file 1 [file Supplementary_file_1.docx]

**周进灵 1153**

*2024年7月17日 下午 10:54
4分钟 50秒*

**关键词**

饮食 习惯 印象 团体 焦虑 领导者 零食 事件 时刻

**文字记录**

说话人 1
嗯，分享一下你在我们这个团体中的一个整体感受和体验。

说话人 2
嗯，整体感受下来是就是挺好的，就是也学到很多嘛。嗯，就是然后在体重这方面也确实减少了很多。

说话人 1
还是很有用的。那我们那个团辅因为一共有 8 次，那你自己的一个感受，就是感受上有经历怎样的变化？

说话人 2
感受上今天怎样的变化，就是最开始其实就是有一种就是不太确定，就是值不值得来嘛？嗯，然后到后面，然后再到后面，最后就是确定就是很值得来的。

说话人 1
嗯，在刚开始的时候比较忐忑，对，不怎么相信，对，嗯，好。那在就是我们团普当中有哪些事件给你留下了深刻的印象呢？世界或者时刻或者分享的东西。

说话人 2
我印象最深的就是给那四个拳头，对，那个比较理想深，然后还有就是正面吃饭。

说话人 1
那个，这个事情对你有什么影响吗？就是我们印象最深。

说话人 2
的就是这个，那四个拳头，就是因为我印象很深，然后后面我吃饭就是几乎就是按照那个量，然后去吃就实施了，是吧？对，对，就是然后现在，包括现在都一直在，就感觉形成了习惯那种。

说话人 1
那我们这个团服在运动和饮食方面有给你带来怎样的变化？

说话人 2
嗯，饮食上面变化挺大的，就是不会再吃小零食。嗯，在其他时间，然后运动上的话就没有太多时间去行动实施，然后就运动还是按照之前以前的样子。

说话人 1
你有没有过情绪性近视的行为？

说话人 2
没有。嗯，对。

说话人 1
那你在我们就是这个团府前的一些期待进来之后有得到满足吗？有的，具体大概是哪些呢？

说话人 2
就是在我的行为习惯上面有一些变化，然后也让我更健康的去进行饮食嘛。

说话人 1
那你在我们这个团伙过程当中，你自己有付出哪些努力来帮助自己实现自己的减重目标？

说话人 2
嗯，就是按照就是团妇留的作业，就比如什么打卡，然后记录饮食习惯。

说话人 1
这些，就是按时完成作业。对，那在你现在就是如何评价你自己的一个状态呢？就比如说是消极、积极还是焦虑程度。

说话人 2
这些状态。没有焦虑程度就还是很积极的。

说话人 1
那，嗯，你觉得你现在的在团组中的这一些变化是哪些因素造成的？

说话人 2
哪些因素。

说话人 1
让你发生了一些改变？

说话人 2
有很多，包括就是老师还有领导者给我们的一些建议，然后还有我们的小组，嗯，团员给我们的一些就是互助。

说话人 1
嗯，好。那你觉得我们这个团组对你最有帮助的地方是什么？

说话人 2
最有帮助的地方就是让我有一个记录饮食的习惯，然后知道就是自己吃的东西热量。什么。

说话人 1
你觉得我们团体最大的特点是什么？

说话人 2
最大的特点健康，哈哈。

说话人 1
你在这个团体中有哪些遗憾没有完成呢？

说话人 2
遗憾就是最开始我们，嗯团服的顺应，那包括我有几次请假的情况就没有完全的参与进来吗？

说话人 1
那如果你要给你身边类似的有减重需求的同学推荐我们这个团服，你会怎么推荐？

说话人 2
我会，我可能就会给他们推荐那个记录饮食习惯的那个APP。哦，对，因为我觉得那个挺有用的。

说话人 1
就是你怎么跟他推荐我们这个团斧？就是让他加入的话，你会怎么推荐？

说话人 2
嗯，让他加入的话怎么推荐？就可能会跟他说在你的习惯上面可以给你很多帮助，然后可以改变，可以让你更健康的，然后进行饮食，包括有规律的运动。
