# Supplementary material for: Helpful factors of group cognitive behavioral therapy in overweight and obese college students
Source: Front Psychol. 2025 Sep 12;16:1585765. doi: 10.3389/fpsyg.2025.1585765 (PMC12463828; doi:10.3389/fpsyg.2025.1585765)
Supplement: Supplementary file 2 [file Supplementary_file_2.docx]

**杨萍 8558**

*2024年7月18日 下午 12:20
36分钟 16秒*

**关键词**

团体 知识 认知 心态 情绪 心理 收获 印象 力量 顾虑 身体 安全感 焦虑 答案 大脑 动力 作用影响

**文字记录**

说话人 1
那我们开始好，就是我们团队已经就这么这么长，持续这么传一段时间，然后也做了八次，能分享一下就是你在这个团体中的你一个整体的感受和体验嘛。

说话人 2
整体来说的话，我的感受是从效果来说，用数据说话是非常有效的，因为我确实在这三个月里面我瘦了很多。然后，嗯，我 show 的原因可能首先肯定是团队，整个团体的一个力量，就是每次大家分享，然后相互交流，然后包括一些关于这个减重的知识，这方面的一个增加，让我能够瘦下来。其次是我自己的一个。嗯，正好是因为这三个月我参加了这个团服，我自己也有一个机会可以发挥自己更大的主动性，去主动地了解这方面相关的知识，所以我感觉自己现在对于减中这个事情并没有那么的焦虑，反而是越了解越放松，然后不会那么焦虑，之后反而会放平心态瘦得更多。嗯，我还是觉得非常的感谢这个团服。

说话人 1
什么声音啊？是我是卡了吗？

说话人 2
是。是吗？我不。

说话人 1
知道，年内听着有声音吗？

说话人 2
有啥声音？

说话人 1
就是有个什么一直在动的声音，就是敲击的声音。

说话人 2
没有，你别吓我。

说话人 1
哈哈哈，真的没有吗？没有，这是我的那个是我耳机卡了吗？稍等哈。

说话人 2
我突然有哎，这个在呢。哎妈，吓死我。

说话人 1
还是有诶。那没有了诶，没有了没有了。好好好，吓人啊。好，然后继续，我们一下子。

说话人 2
下了两个人。

说话人 1
然后那就是因为我们团服持续了这么多个月，就时间跨度比较长，那从最开始一直到现在结束了，这个中间就是你的感受有没有什么变化呢？就是在最开始，然后在中期或者一直到结束，你的感受有什么变化？

说话人 2
嗯，其实是有的，而且对于我自己来说变化还蛮大的，因为一开始的时候我可能会有一些顾虑，比如说这个团服它到底有没有用？有没有效？这个团服他会不会帮助到我？因为我在来之前我自己也算是有一定的有一点点了解，大概我就算不来，我可能也会瘦下来，但你要说效果的话不一定会比这个好。然后我来了之后，慢慢地随着自己的投入越来越多，我发现其实每一次团服结束之后带来的那种效果是非常奇妙的，每一次结束之后，我都感觉自己好像充满了动力，可以去有很对自己有很多信心，我可以去把这个事情做得很好很好。嗯嗯，所以这是团队给的一个力量。其次之外是后来随着自己的投入之后，我发现我更就是越投入越有收获，我反而减得更快、更有效果、更信心，所以我觉得这个是非常大的一个变化，后来就很大的一个转变。

说话人 1
那个我今天跟那个明月聊了一下，他也是说就是团体，就尤其是你在群里跟他互动比较多，也给到他很多帮助，就是互相的一个力量。

说话人 2
对，因为其实减重这个是知识点，之前陈娟老师也有讲过，就是根据什么数据表明世界上减肥成功的人也就那么7%，其实不多。那还挺少，成功减肥他肯定是你瘦了之后你能保持的有7%。嗯，然后瘦了之后反弹的也有一部分很大，但是现在我们还在，你能不能售这一个阶段。

说话人 2
嗯嗯，对，虽然说售了之后能不能保持这个事情可能大家都想保持不一定，但是我觉得如果说它的数据是7%，说明了它其实是很难的。那如果在这个阶段可以相互地提供一些支持，你去帮给到一些力量，其实你在给的过程当中你自己也会收获很多，因为你给了别人，下一次你发的时候别人也会给你。嗯，所以这是个相互的过程。嗯，对，他有收获的同时我也有收获。

说话人 1
嗯嗯，那你觉得在这个团体中有没有哪些事件给你留下了比较深刻的印象？对，你现在能马上想起来的比较深刻的场景。

说话人 2
一下子我想到的是赵老师讲的一个知识点，就是那个我查吗？我是那个恶感和壳感的那个神经露出挨得很近，所以你感觉到恶，给你感觉到渴，他两个感觉神经中枢是挨得很近的，有的时候你可能想吃东西，那其实是，那可能不是你想吃，是你渴了，就有的时候比如说我们吃饭很干，你就比如说你吃咸的，你就很容易继续吃下去。

说话人 2
如果你没有喝到水的话，其实你是想喝水的，但你又一直吃，嗯，所以比如说你要去分辨一下你到底是渴了还是饿了？这个知识点当时一下子就进了我的脑子，然后一下子是这个，其次就是还有一个是上次你和雨婷姐没有来的那一次还蛮。嗯，印象深刻的，因为那一次除了你俩没有来代理，没有你俩的作用，没有你俩之后真的这个很不一样。除了这个之外，那天又加上了天很热，换了一个教室在三楼，然后又没有空调，又很闷。嗯，空气也不清新，就是那种没怎么用我们的教室全部 buff 点满之后，真的当时就脑子里面所有人都有一样的感受，就是好想离俩回来。

说话人 1
真的吗？

说话人 2
哈哈哈就就就真的是那次大家都觉得好像缺不就是平时可能还没觉得说。嗯，有什么，就那一次生深刻地体会到了你和于婷姐的一个重要性。是这两个。真的。

说话人 1
吗？我们现在都觉得我们自己可有可无的。

说话人 2
真的要经历了体会了才会，因为其实在团服里面大家都会发现，嗯，来的参加的同学大多数都还比较的内向和自卑敏感。嗯，就有的时候不是说我给到他们一些建议就有用，因为他们要的可能有的时候不是建议，是你给他一些心理、心灵层面、精神层面的一些安慰、鼓励呀。

说话人 2
嗯，这些可能是他们更需要的，所以有的时候这个认知方面的改变不一定说一定要是知识方面，而是对他们对于自己的看法，对于我敢不敢去说出一些事情，比如说我的在减肥中遇到的困难，其实我肯定能相信，在团队里面，我当时那天也看了有一些同学，他是没有变化，体重其实不多的，也有。

说话人 2
嗯，那他们肯定是有很多很多疑问的，但是，嗯，每次一到分享的环节，其实大家都不太会，不敢有的时候，就比如说有的时候我还是会有一点觉得感谢大家的包容的，同事又觉得有点歉意的是我很想活跃气氛，但是我知道我用的方法并没有那么好，因为我只是用了给大家传输一些知识方面的东西，但有的时候他们需要的并不是这样，我给的知识越专业，反而对他们来说是他会觉得你离他很远，然后会给他一种我好像更不行。我好像更不好的感觉，他就更不敢去说了。嗯，或者说碍于场面他更不想说，所以这个也是为什么每次有的时候有些同学他没有办法开口，我觉得这个也是一个原因。

说话人 1
但是就你的角色，你本来也不是一个代理者或者协同代理者，你已经做的做得很好了，然后就这部分就是心理上的这部分的一个抚慰，肯定是主要应该是由我们来做的，你也不用就是歉意什么，因为也今天我跟其他同学也访谈了之后，他们也有很多就是表示，就是对你说的那个，你那个果茶，是你说的奶茶和果茶那个热量，他们也觉得也就是印象深刻的，他们觉得这些，你传授这些知识对他们印象也很深刻，就是很有用。嗯，也是有成员会觉得有用的，只是每个人不一样，像觉得就是心灵更需要抚慰那一部分成员肯定这部分的责任应该是由我们来做的。你也不用就是感到怎么。

说话人 2
没有。我倒没有，只是说会觉得如果能做得更好当然更好吧。

说话人 1
我经常在跟雨晴说呢。我说你比我们两个更像协同，更经常。

说话人 2
笑死。不不不，我只是在补充我，我们仨一起加油，哈哈哈。嗯，主要还是你们因为这个它过程你们俩要cue，有的时候比如说尴尬了或者说冷场了，就只能你们来，因为这个角色是你们两个在一直在带领的，有的时候没办法。真的辛苦你们了。

说话人 1
你也很辛苦，我想说真的，你很像协同在你这。笑死啊，那你觉得你对刚刚你说的这些事件的一些你的具体感受是什么呢？你的具体感受和反应就是刚刚你说的这些深比较深刻的事件，那你对他们的一些感受和反应是什么？

说话人 2
感受的话，其实我会觉得我也有经历过像他们这样的一个阶段的，就是不敢去表达的一个阶段。或者说我很害怕被别人发现，我会去隐藏，我也有过这样的感，这样的一个体会，所以我能理解。但当时在那个场景下，大家敢说的这个这种心情，嗯嗯嗯。但是我还是觉得可能我也不知道怎么应对。

说话人 2
其次是我觉得可能这个团辅可以，你如果说以后还会开展的话，可以加一点那种就是一些活动的东西在里面。对，就像自，你比如说提升自信，你每次都可以有一个动起来的，就可能不多，就一个也可以就动一下，增长一下大家的一个自信心。嗯，因为很多时候不，除了认知，有的时候这种积极心理学方面有的东西确实还是很有用的，包括就是第一次，我现在都记得第一次团体辅导结束之后，我们不是每个人都用一句话来总结就。嗯，当时是有个同学，他就说到现在无聊。嗯，对，他就说有点，好像有点无聊。是第一次来，他说嗯，也没有动，好像也感觉有点像是快睡着了，然后后面发现好像每次都这样都不动。嗯，而且第一次我们还有一个活动，是那个记左右伙伴传这样子的那个。对，还有几个活动的那种可以稍微活跃一点的，你看就是后面从第二次到第八次，我们都没有一个可以具体的一个活动的形式来帮助到大家。

说话人 2
嗯，可能最多的就是第一次和好处回应卡，所以这方面如果多一点的话，可能对于同学们那种内向不敢说，可能要好一点，比如说他不敢说，他可以写下来，我们匿名放到里面，我们就打开互相分享，这样都好一点。就比如说我有一个疑虑，就是这个平台期的时候我怎么办嘛？我不知，我可能自己有一些心得，但我不知道正全面地去看，那我写了这个放进去，抽到了，我可以主动地第一个说出我知道的那一点，然后其他同学一起说，那这样其实进行的会比直接我那种大家坐在一起表达的那种风险，可能对他们来说要承担的要少一点，我觉得这个是可以考虑的。

说话人 1
这个问题我们等一下访谈完了我再跟你细说，哈哈，然后下一个就是，嗯，你觉得我们团体在运动或者饮食方面，这两个方面给你带来了哪些变化？然后这些变化对你有哪些影响呢？

说话人 2
运动合影是，嗯，有的。嗯，因为我其实饮食的话我以前也知道说减肥我应该怎么吃。嗯嗯，但是更多的一个变化就是执行。嗯，因为很多时候干就完了，你知道什么并不重要，你知道的多。怎么了？你不做那还不是等于没有？所以对我最大影响就是我获得了一个动力去做，而且一旦我开始，我就不会停，我就一直开始了，所以这是最大一个变化。然后运动的话也是因为。嗯，可能我瘦的时候我运动就运动嘛，我就去跑、走、坐，我啥都不管，但是你胖了之后你会有还是会有一些害怕被评价等等的一些顾虑在你，可能，然后包括你胖了之后，我自己也说到我的这个身体没有之前那么好了，我可能腰酸，我可能身体真的没有之前跑步跑起来那么轻松，会累，会很不舒服。那我现在运动就是慢慢地去加，放平心态地慢慢做，他就慢慢地去做。所以我觉得更大的变化是执行和长期的一个心态的平稳，让我敢于去接受。比如说我第一次运动完了之后很不舒服，但是我也不会就放弃。嗯嗯嗯，没关系，这就是一个那种啥呢过程，它是一个过程，我感觉就是这样子。

说话人 1
嗯，那你之前在吃的方面你也很有很多了解哈，那你有你是怎么看待就是情绪性进食这个问题的呢？

说话人 2
嗯，我感觉可能胖，除了就是从小的一种可能，一直是一样的饭量，然后一直胖的同学，你可能突然胖，或者说一下子胖，其实大多数都是因为情绪性侵蚀。嗯，以前我会觉得这个是一个很少很。嗯，是，就是没有那么多人会议的，后面我才发现其实胖就是肥胖，你胖了很多都是因为情绪性近视，所以这个好像它很普遍。

说话人 2
首先是我，我看到了它的普遍性，其次是以前我也一直看到这样的说法，我不下看到 50 个帖子。 100 个帖子都说什么你吃食物不等于爱，我以前对他的感觉就是我知道食物不是爱。嗯，而且我很清楚。后来诶我是最近突然有了一个感悟，是在我进行正念饮食之后，因为正念饮食的时候我在品尝那个食物的味道，我就知道它什么味。但是你情绪进食的时候你是不知道它什么味道的，你只是在吃，嗯，你只是享受。而且你享受的只是吃进去的那一瞬间，你脑子分泌的多巴胺的那个快乐，就是你享受的不是食物带给你的快乐，你享受的是吞咽的那个过程。嗯，对大脑的刺激带来的快乐，而那种快乐其实更像是一种毒品，就是不断地希望一直吃，一直刺激大脑的那个神经，一直达到快乐。但是你的嘴巴、你的身体、你的胃都不快了。所以情绪禁进时像是用伤害自己身体的方式去满足大脑，这是我的感觉，所以就是你说。

说话人 1
请专业，哈哈哈。

说话人 2
真的是我的一个感觉。然后，嗯嗯，如果说有情绪性近视的话，慢慢来，就是看到自己为什么会情绪性近视？原因是什么？是焦虑吗？是迷茫吗？还是说你仅是因为你吃碳水吃上瘾了还是怎么的？就是找到那个原因去消化它？如果你需要一段时间才能够把情绪继续进食去掉，那没关系，你可以给自己这一段时间，在第一次你可能做不到把它完全戒掉，那你就慢慢来，就慢慢地经过四次、五次，你可能后面你就戒掉了，所以这个东西是一个慢慢改变的过程，而且它这个很难的过程，因为它不是身体的，它是整个人心领的，包括安全圈感等等那些东西，所以它是个难的东西，我感觉是需要给到。怎么说呢？如果说真的是有一个来访者，或者说有一个人来求助我的话，我可能会，我只能说不，我可能给不了他太多，我只能给到他心理层面的一个安全感，因为他可能需要的也是安全感。

说话人 1
对，没错，这个应该是更重要的一会儿。哈哈哈，下一个，哈哈哈，那就是在一开始进团体之前，你肯定是有一个期待的。那我们现在团体结束了，你觉得你在开始的一个期待有没有得到满足？

说话人 2
嗯，一开始是有期待的，因为上胡老师的课的时候，对于团体辅导我的出印象还是以活动为主嘛。然后虽然这次团体辅导的名字叫做认知减重，但我对他的定义是有认知，有活动，就是相加，这个认知减重就是一，就是在活动中改变认知等等。可能对它定义和来了之后得到的定义会有不一样的地方，但是整体来说是可以 10 分的话可以打 8 分。被打 8 分是一是满足了我基本的一个期待，因为我确实通过这次团腐，不仅是数字上面受了，还是我整个人的一些心理层面的一些完善和一些丰盈，这样的话可能我在未来可以受得更多更持久。嗯，那你姐姐，你。

说话人 1
你说怎么了？没有。

说话人 2
我只是想吐槽一下这句，别剪下去，我说你后面剪我的肯定很复杂。唉，辛苦了。

说话人 1
没事，只是转文字做质性分析。好的，那你觉得哪些有？嗯，没有满足呢？你说基本得到满足，那有没有满足的地方？

说话人 2
其实不算是满不满足，是期待有偏差，因为之前我的理解的定义和不一样，来了之后的定义不一样啊。然后另外一个期待是，我可能之前以为两个月结束，我可能会受很多很多这样子的一个状态，但是可能有一之前我的想法也比较的有点理想主义，或者说，嗯，会有一点理想主义，嗯，然后，嗯，这样之后我来了之后才正好是把我的那种想要追求快速减下来，这种心态变了，转变了，所以，嗯，其实是不存，不算是不满足，只是说期待的东西有偏差。

说话人 1
那在这个过程中你虽然就是可能没有达到你一开始想要瘦的那么多斤，但是我看你还是瘦了挺多的。那在这个过程中你做了哪些努力来帮助你实现你这个目标？

说话人 2
瘦的话首先是团体辅导，它本身就有一个督促作用，你每天对每个周都要上秤一次，嗯，你上秤如果就是等于上行吗？嗯，哈哈哈，就一上去你就得看到那个数字，你就，而且我来的目的就是为了减重。嗯，所以我是目标导向，他做计划，比如说我这个周我要瘦两斤，那我瘦两斤我需要做什么？我要怎么做啊？我能不能做到？我会给一个计划，然后去评估一下这个计划能不能实施。我就做如果中途遇到什么特殊的，或者说，嗯，不能够按时进行的，那就可调整，但基本上我是差不多按计划执行，差不多是这样的一个状态，就是目标不加计划加行动。

说话人 1
是的。那你这个过程中你自己有哪些改变呢？就是你自身的一个变化，你觉得。

说话人 2
城市改变其实还蛮多的，首先是我对于减肥相关的一些知识肯定是多了很多很多。嗯，除了赵老师，然后包括我自己去了解，知道了很多，不仅是减肥，更多是，那我之前可能知道的是关于减肥和减重。然后我来了之后我了解更多，之后我关于这个健康方面也知道的更多，这是一个其次是关于我任直方他的改变吗？就是长期主义也说了很多次了。嗯，就是放平心态去慢慢搞，然后最大一个点就是我对于这个爱自己有了很大的一个感受。嗯，因为以前我认为的爱自己可能是口号，但现在我好像找到他答案了，但我可能用语言表达不出来。

说话人 1
嗯嗯，那我感觉你已经本来下一个问题是你怎么试了下你现在的一个状态？我感觉你也已经说过了，就刚刚你回答，哈哈，可以可以。嗯，那你觉得发现的这些改变，不管是你自身的还是团体带给你的，反正就各方面的一些改变，它对你的生活有什么影响？

说话人 2
会有我现在生活影响有最大的。我一下子想到的就是我的饮食习惯。嗯，改变了。嗯，因为你了解了很多关于健康的知识之后，你肯定会想要说大家都想健康，但是最重要的是你能不能。

说话人 1
做出。

说话人 2
一些改变。可能之前我想着，诶，我肯定也要健康，但是我垃圾食品或者说重油重盐我也照样吃，因为我并不会因为我想要健康而去做出多大的一个，因为你还没有得到一些知识性的东西，或者说你还没有了解到你，没接触到你，所以你不太懂怎么去做，这也是一个原因。然后现在的话我可能吃得会更加的。嗯，没有那么多条条框框，自由一点，口味会比之前清淡很多，但我不觉得这样的清淡不好，因为我仍然可以吃辣的，吃油的。只是说我变得没有那么的去追求，要辣、要严，我觉得很棒，然后我会额外的每天吃一些身体摄入不到的一些维，那个叫啥？膳食纤维也会去摄入一些，对，嗯嗯，然后就我觉得这是很大一变化。而且我现在不会，就以前我会害怕，就是因为自己吃得清淡一些了之后会和身边的人不太合群，这些一些顾虑在，但是现在我不会，我就这样吃。嗯，只是去平衡掉这一部分的顾虑。

说话人 1
那你觉得这些变化是由哪些因素促进你成长发生改变的呢？

说话人 2
嗯，首先是肯定是团体辅导给了我这样的一个机会，当我在团体辅导的时候看到自己瘦了，我就很开心，然后又因为也是互联网的作用，互联网一直推相关的内容知识，我又去有这个机会可以去了解到很多科学性的，包括不管是在书上还是论文期刊上面的一些东西去了解才让我有了一些变化。就是一，就是它不是一个叠什么呢？它不是 1 + 1 大于 2 = 2，剩余加 1 大于2，就是各种加起来一起变得更好。也都有，大家的都有，就包括团队每一个人我自己。

说话人 1
都有。嗯嗯，好。那你觉得，嗯，这我们团体对你的帮助和作用影响有哪些呢？

说话人 2
团体对我的帮助作用和影响首先是一开始的一个最明显的影响是每次结束之后都会有个一个奇妙的一个动力增长、信心增长的过程。嗯，可能我说到有一些同学他不敢说自己什么什么，但是这些虽然有一有这样的问题存在，但是你不可否认的是每一次结束之后真的会每会有一些奇妙的变化，这是团体给的一个很直观的，最直接的一个变化，其次是认知方面，大家一起相互讨论一个团体的力量，包括赵老师给的一些认知识什么的都很有用。嗯，然后对我影响，还有就是对于这个特点的团参与的人群来说，我们的带领者和协同带领者应该怎么去做？怎么去带动、怎么去搞，其实是一件蛮需要积累，或者说需要下来认真去思考和复盘的东西。 ChatGPT 不是一下子就能说我要。

说话人 1
马上发生改变。十分。

说话人 2
对，你，你真的得。

说话人 1
积累的过程。

说话人 2
对，而且每次你要根据临场的一个去变化，因为每次的那个。

说话人 1
遇到的情况不一样。

说话人 2
冷场的原因也不一样什么的，所以这个也是需要我们去慢慢锻炼的地方，然后更大的影响是我自己变得更心态好了很多。

说话人 1
那你觉得最有帮助的一个地方是哪一个最有帮助？

说话人 2
那最有帮助的肯定是对于心态方面的变化，因为心态变了之后你才能走得更远，所以但你要用最的话，其实都是这个心态变化，也是在其他的一些变化的基础上做。嗯，产生的或者说运作的我觉得都很有用。

说话人 1
那你有没有什么遗憾呢？没有实现的。

说话人 2
遗憾。

说话人 1
就可能是会赢，会有。

说话人 2
遗憾的话肯定还是会有，因为其实每一次结束，每一次我们都是新的体验。嗯，去之前来之前开始，每次之前你肯定都有一个嗯想法，或者有，比如说我这次要说我有一个预期，对，然后开展的时候有各种各样不同的一个场域，不同的进行，结束之后又有不一样的收获。其实每一次要说遗憾的话可能就是。嗯，感觉并没有把这个团服里面大家的一个状态待得更好，虽然我不是协同，但领着，但是我天生会这样想，有一种这种我会这样责任感，可能是我本来就是，比如说我一直就是一个喜欢去调节气氛的人，但是这次我没调节成功。

说话人 1
就有时挫败感。对。

说话人 2
然后我会觉得有遗憾在这一点是我的遗憾，其次的遗憾就是可能。嗯，有一些问题好像并没有给到同学们一些解答，嗯，可能我知道答案，但是我可能说出来就。

说话人 1
变样了，嗯。

说话人 2
不太好，因为一下子，比如说他，其实他要他说他问这个问题不是要答案，他要的是安全感，他要的是安慰。嗯，所以我不会给到。那你其实他有一个心里面可能有一个，有个小人物也在说他其实也想要答案。嗯，对，这也是一个遗憾。

说话人 1
嗯嗯，那总体来说你觉得我们团体最大的特点是什么特点。

说话人 2
是吗？对哦，最大的特点是大家都挺迷茫的，但是都很坚持。我觉得是怎么去说呢？就是首先是好像我们都是第一次做，哈哈哈，是吧？赵老师？我不知道，反正。

说话人 1
他不是，他好像第三次wow，看不出来是吧？

说话人 2
这可是可能是，我感觉其实我挺迷茫的，然后丹杰和雨婷杰好像也是第一次当启动带领取了。嗯，其他全员也是第一次，大家都迷茫，然后但是都还是坚持下来了，每一次都还是来了。嗯，还是在努力地。

说话人 1
去完成，他。

说话人 2
做到有有帮助到别人，有也有帮助到自己这样子去做。所以我觉得这个还蛮。

说话人 1
你形容得很好，坚持迷茫。

说话人 2
真的不容易。

说话人 1
大家都好。最后一个问题了，如果你要给类似就是有减重需求或者有情绪困扰的同学推荐我们团体，你会怎么说呢？

说话人 2
有情绪困扰。

说话人 1
就类似的困扰或者有减重需求的可能情绪块就是他们，就。

说话人 2
我要向他们推荐。

说话人 1
对，嗯，你会怎么说？

说话人 2
要推荐我们团服？就是首先是可以尝试一下，试一试认知团服是什么样子你可以知道，其次是你在这里可以收获一群志同道合的伙伴。嗯，你也可以了解更多关于减重方面的知识和需求，更重要的是你可以从心理学的角度去看待减重。嗯，解决你在心理上的一些困扰，比如说给你科普一些心理学方面的，比如说情绪性进食呀等等等等等等，这些肯定是大家都很想很想知道的。嗯，好。

说话人 1
我们好的，结束了。

说话人 2
好的，你关录音吧。

说话人 1
好，马上。
