# Supplementary material for: Helpful factors of group cognitive behavioral therapy in overweight and obese college students
Source: Front Psychol. 2025 Sep 12;16:1585765. doi: 10.3389/fpsyg.2025.1585765 (PMC12463828; doi:10.3389/fpsyg.2025.1585765)
Supplement: Supplementary file 3 [file Supplementary_file_3.docx]

**王佳欣 1321**

*2024年7月17日 下午 10:54
5分钟 57秒*

**关键词**

团体 饮食 焦虑 分享 热情 活动 交流会 冲突

**文字记录**

说话人 1
请你分享一下你在我们团体中的一个整体感受和体验。

说话人 2
那我觉得团体就是团普的活动当中是很放松的。然后也就是也不会有，比如说我之前来之前会感受，会，可能会想象的有一点，嗯，就是没有体会到会有不舒服的感觉。然后，嗯，跟同学们的交流当中我也可以有，就是能感受到他们的热情。嗯嗯，我觉得是。

说话人 1
那就是因为我们团普经历了 8 次，那你中间的一些感受有经历怎样的变化吗。

说话人 2
其实一开始就是第一次，第二次那段时间我的热情还是很高的，但是到中间的话可能会有一点懈怠，然后因为跨度比较长，然后到最后几次的时候可能也会有一点点，嗯，懈怠，然后特别是后面的。嗯，各种活动，嗯什么加起来也会比较有可能会把时间有冲突，然后就可能有几次没来，然后那几次没来的话就会可能也会有点懈怠。是整体我觉得。

说话人 1
还是可以的。好，那我们团团府当中有哪些事件给你留下了深刻的印象呢？

说话人 2
那个正念。

说话人 1
进食了。对，那你当时就是对这个的感受是什么呢？

说话人 2
因为我之前吃饭的话其实是比较快的，然后有点囫囵吞枣的感觉，然后但是那一次就是老师给了个那个葡萄干，你这个第一次吃到葡萄干那么香甜。

说话人 1
好。那这个事情对你有什么影响吗？

说话人 2
嗯，我觉得他减慢了我之后的生活中进食的速度。不会，我是突然想起来尝尝这个东西到底是什么味道的，我可能会慢慢地去品尝。

说话人 1
那我们团辅有在运动和饮食上给你带来变化吗？

说话人 2
那个吃东西的时候，那个吃饭的时候一拳，两拳，那个那个我是有在专门就是吃饭的时候注意到。

说话人 1
嗯，就是饮食的量会变少一点，对不对？嗯，那运动上有什么变化？

说话人 2
运动其实我因为我比较不喜欢运动。嗯，我就没有怎么运动。

说话人 1
那就是你饮食上的变化有给你带来怎样的影响吗？

说话人 2
嗯，吃的变少了。嗯，可能对体重有一点。

说话人 1
影响，你平时会以情绪性近视吗？嗯，很少。嗯，那你在我们参加我们团体之后，你的一些入组前的期待有得到满足吗？

说话人 2
我觉得有，因为像我就之前就说想要更多了解一些关于这方面的知识，然后。

说话人 1
嗯，这个，嗯，好。那你在我们这个过程当中你自己有没有付出什么努力来帮助自己实现减重目标呢？感觉还是。

说话人 2
有，就是吃零食也变烧了。

说话人 1
那你如何评价你现在的一个状态呢？就是你的是消极、积极还是焦虑程度？这些我觉得我还。

说话人 2
不够积极。嗯，但是很焦虑的话，其实我感觉我现在的心态还好一点，没有之前那么焦虑。嗯，就是可能会采取一些，就是完全不吃东西的那种不太好的方法。

说话人 1
来让自己剪中。好，那你觉得我们这个团体当中最有帮助的地方在哪里？

说话人 2
就是关于饮食上面的，还有我的一些思维方面的变化。

说话人 1
你觉得我们团普最大的特点是什么？感觉是。

说话人 2
嗯，就让我感到安心。

说话人 1
你在我们这个团服中有哪些遗憾没有得到满足吗？

说话人 2
遗憾，但是好像。

说话人 1
没怎么。嗯，很完美吗？

说话人 2
可能后面来。

说话人 1
的比较。

说话人 2
少，是吧？对，然后中间有这部分，也是一些同学也是请假嘛？嗯，可能，嗯，每个同学之间的交流会比较少一点。

说话人 1
那你觉得未来就是我们这个团服有哪些方面可以做出改善吗？嗯，我觉得跨度可能有一点太长了，就是中间那个时间。

说话人 2
对，特别是到期末这段时间的时候特别忙。

说话人 1
那如果你有给类似检送需求的同学推荐这个团体，你会怎么说呢？嗯，就是怎么给他推荐。

说话人 2
我觉得可以在这个团体中找到很多有同样困扰的同学。嗯，然后在平时的分享当中也会互相抱团取暖，然后互相帮助之类的，会给我一点信心。

说话人 1
好，那今天的那个就到这。
