# Supplementary material for: Helpful factors of group cognitive behavioral therapy in overweight and obese college students
Source: Front Psychol. 2025 Sep 12;16:1585765. doi: 10.3389/fpsyg.2025.1585765 (PMC12463828; doi:10.3389/fpsyg.2025.1585765)
Supplement: Supplementary file 4 [file Supplementary_file_4.docx]

**彭思思 4225**

*2024年7月18日 下午 12:19
13分钟 58秒*

**关键词**

知识 团体 饮食 肌肉 爸妈 体重 心态 认知 焦虑 素质 成果

**文字记录**

说话人 1
好，那我们先就开始了，首先就是你在这 8 次团体我们已经做了，然后在这个团体中你的整体感受是什么？还有你的一个体验。

说话人 2
整体感受就是，嗯，大家的谈论还他们之间，我们之间的讨论还是很和谐的，嗯，最主要就是大家能够分享彼此的一些经验，然后再可以说是抱团取暖，然后一起前进吧。知道是有人督促我一起在这个简中山一起走吗？

说话人 1
就是志同道合的一群人在一起，然后你共同努力。对对。嗯，那就是从第一周一直到第八周，其实我们这个也是经历了很长一段时间，那在这段时间里面你的感受有没有什么变化呢？就是从最开始一直到现在，我中间有经历哪些变化呢？

说话人 2
嗯，最开始的话我还是，嗯，我本最开始可能是抱着一些有一点那个功力，就觉得参加的东西肯定还是和，就是非常注重素质的变化。然后到后边我是我的第一个追求气质，都是跟大家一起聊聊天，然后学习一些基本的知识，然后想都是以后我慢慢来的感觉。嗯，又没有非常功利了，主要还是追求一下心灵的一些提升。

说话人 1
嗯，就是心态方面发生的。对，改变。嗯，那在我们这个团体中你印象最深刻的事件有什么？就你现在一想起来，哇，马上就能想起来的，非常深刻的是事件或者场景。

说话人 2
罪是个责，还是我的奶茶这么多年的白喝了啊。

说话人 1
哈哈，你就是一个知识，是吧？对对对，果茶和奶茶是吧？对。

说话人 2
对对，都白喝了。以前非常自作聪明，觉得自己嗯又喝到了，然后。

说话人 1
又不会胖很多，这个结果发现果茶更多。对，更夸张。嗯嗯，那你在知道了这个知识之后，你的具体感受和你的反应是什么呢？

说话人 2
确实不下来，不太想喝果茶了。哈哈，很就已经不是不喝奶茶。

说话人 1
了。这可能还需要，我们也需要，就是在我们减重过程中也需要去了解更多的知识来帮助我们减重，而不是就是自己就一股资金就在里面，结果也没有收到什么成效。对对对，嗯，那这些事件对你有什么影响吗？

说话人 2
也就是不能自以为是的觉得一个是他的一些知识嘛。嗯，像我以前觉得果茶这边奶茶健康，然后还有什么，反正一系列的错误的认知吧。嗯，就像我以前认为锻炼之后肌肉会尊重人，然后自己重量会上升一样，其实它是个长期的过程。我如果但凡在这个期间自己上网查一些东西，或者是询问过，又不会这样子一直错下去，然后后面还非常觉得自己以前傻傻的，而且这些钱好像也白花了。

说话人 1
嗯，就是改变自己的一些错误的认知，让自己能够更健康的减重。对，嗯，那刚刚就我们可能谈到的比较是饮食方面的，那你觉得在这个运动方面给你带来哪些变化呢？

说话人 2
运动方面的话，嗯，我以前觉得要减肥的话，就必须是长期剧烈的时候，比如说那些网上什么帕帕梅拉，然后要跳绳。跳多少个呀？嗯，那现在我突然觉得其实也没有那么需要，我一周来个一两次，然后也不需要一定要跑步，一定要跳很剧烈的。神，我只要平时饮食健康一点，想出去有时间就去走走两跑两圈，或者是跳过绳，没时间就去走两圈，其实也还行。嗯，不用的，给自己那么大的压力，因为我以前觉得只要自己没有坚持那样高强度，感觉就是白锻炼了一样。

说话人 1
那对于情绪性近视，你个人是有这个情况吗？

说话人 2
嗯，我没有情绪性时代。

说话人 1
那你饮食方面的变化，除了刚刚我们聊那些，还有其他的吗？

说话人 2
嗯，饮食方面的变化，其实我平时就不怎么吃那些其他东西，从因为很早以前，对，很早以前我就想减肥，我爸妈捞了很久。嗯，所以我没有系统的去，因为我很懒，我觉得影视这个东西是最不费力的。嗯，所以我就从可能参加团会前一两个月我就已经开始不吃影视了。嗯，我觉得还好。

说话人 1
那就是我们现在团体已经结束了，你在。嗯，进入我们团体之前肯定是与对我们团体有一些期待的，那你这些期待有没有得到满足呢？

说话人 2
嗯，总体上都得到满足，虽然我最后的成果因为我个人的原因好像没有很明显，但是在团普，我不管是从那个人际关系，那些心灵上的一些交往还是一些知识，然后我觉得我的减重还是有成果，毕竟前两次的体重还是在掉的。虽然后面回来了，但是所以说我觉得团伙还是在我心理上，虽然没有我最开始期望的那么高，但是还是有一定回报的。

说话人 1
嗯，那你觉得哪些是没有达到你的那个目标？或者说你有一些遗憾的呢？

说话人 2
也就是我这个人太懒了，本来开始一两周还是严格执行什么要拍照，什么时候锻炼一下。嗯，到了后面还是有点惰性，除了最基本的什么饮食，那些东西能坚持下来。但凡是运动那些东西还是会懒惰一下，所以后面的成果就不可能不是很好。如果我以后能够真的有什么下定决心，比如说我妈的刀架脖子，然后冰雪对我可能才能真更好的运用这个知识量，真正达到减重。嗯，就。

说话人 1
还需要有动力，对外有外部的动力来推动你去来完成自己的一个减重。那刚刚谈到可能有些方面你的动力可能会不足，但是你肯定也是自己也做了一些努力的。那你具体是做了哪些努力来帮助你来健康健植物。

说话人 2
一个就是本身食欲不是很强嘛？然后我也很少，主要是我的努力的话。

说话人 1
就可能主要是在饮食方面就控制一下自己的饮食。

说话人 2
我就是因为是一个虽然功力不是很大，但是我也是个非常的算踏实的人，比如说要老师要求上体育课，我的我基本上都不会怎么偷懒，然后在饮食方面的话，因为本来我是那种不喜欢吃东西，但是吃会吃很多的人，嗯，所以我只要在最源头上不买东西，我就基本可以杜绝食灵是这个事情，嗯，所以说我就每次就我就不喜欢上那些。不上那些购物软件，然后也不跟他们出去，每次他们让我去北屋买吃的，我就以懒为接口，我就不去，然后我就可以不吃这些东西。

说话人 1
那自己还是我感觉这个也是很需要自制力的。哈哈，控制。对，嗯，那一直经历到现在，你觉得从最开始到现在你有什么变化呢？你。嗯，自身有哪些改变？还有你对自己的看法有没有改变？

说话人 2
我以前对我自己的体重是有点焦虑的吧。嗯，因为我作为人证都成功减重了。嗯，然后我爸妈也一直在催我，然后网上还有很多那些视频。但总结很焦虑，就像我加团伙之前那两周，我真的是每周都明明都上课都很累了，还要出去跳绳，然后做一些什么操，真的很累。然后到现在可能知道一些健康的减重，我就反而心态平和了。嗯，就没有非常的追求，一定要非常非常瘦。然后短时间瘦了多少斤？我一直觉得我这体重其实微胖。嗯，减就减了下，减不下来。只要健康一点，匀速下来也还行。嗯，所以说就没有那么焦虑了。就确实，也可能也是给我最早的借口，反正就是对于生态教育这方面要稍微好一点，可能就是大家都一起在分享自己的事情。所以说还是交流最重要，心里有什么什么不好的想法还是要说出来比较好，不能自己一个人闷着。嗯，像我如果把这些事情跟我爸妈说，可能他们也不会那么逼着我。

说话人 1
嗯，对，那有去跟他说就感觉，嗯。

说话人 2
主要是，嗯，你怎么说？因为我爸妈真不怎么后悔，他们都是跟我那种半开玩笑的，半夜认真的跟我说，你再胖你就成我们家最胖的那一个人。然后那。

说话人 1
你家你都是很瘦，以前本身也不是。

说话人 2
很胖啊。我以前我们家以前都挺胖的。嗯，那我弟因为一直被打击，从小被打击到大，每次出去他们都在说他，然后他就非常的暴躁，而现在就开始卷中，现在感觉他都快成一个那个大哥大，我就装那个。

说话人 1
感觉就是练很多肌肉那种。

说话人 2
因为看起来也不至于到那种肌肉的程度，但就是看去紧实多了，然后好像这么多年坚持下来也瘦了快四五十斤了。噢，那还是很成功。所以对比之下我爸爸就给他看我哪都是颜色，我最坚持不下。那坚持不下去。

说话人 1
那人竟确实，但是男生跟我们女生构造不同，他们确实更容易减一些。这很正常。就我刚刚你说的肌肉就确实，虽然肌肉会重一点，但是我们如果练的一些肌肉，把相应的脂肪变成了肌肉，肯定自己的线条会好看一点。就算体重你可能没有太大的变化，但是你整个人看着就是瘦了，就会好看一点。那你觉得就是你刚刚阐述的发生了哪些变化？你觉得这些变化是哪些东西来促进你发生的这个变化呢？

说话人 2
可能就是知道的多了，见的多了，然后心境就平和了嘛。嗯，以前就是在被局限于那个妈妈的一些观念，然后一些朋友一些调侃，听多了就觉得真的好像自己好像真的有点胖。嗯，然后你走出去看到一些很大家跟自己有同样经历人，他分享自己的经历。然后说一些自己觉得好的一些方法，这些东西，然后你放开了之后知道一些健康的方法，然后也知道自己好像也没有他们说那么胖，嗯，然后也不是那种肥胖，好像也没有照他们说的那么严重，然后心态平和了之后才能真正的才能去减重，而不是过度的那个去一些做一些不好的事情啊。像我最近不像以前刷了很多什么什么肥胖，倒刷了很多减重过度，然后一些不好的影响就是只有自己放开了才能看到更多的信息。

说话人 1
就先接纳，然后才能自己才能更好的去完成自己的一个目标。嗯，那这些变化你觉得对你的生活有哪些影响？还是就像刚刚那个更大，如何？对，那你觉得有哪些因素来促进了你的个人的？就是这么一个成长？

说话人 2
嗯，因素，一个是上了大学之后好像觉得体重已经是一个最不可一提的一个压力了吧。嗯，然后还有就是一个是自己在当下环境下已经觉得没有必要一直局限于这个东西了。然后还有也是我前面一直说的就是有一个更好的宣泄的点，和一个发泄情绪一起吐槽一起进步的一起。

说话人 1
就是提供了这样一个平台。

说话人 2
对，还是只有你说出去了才会平衡嘛？一直憋着也不好。嗯，然后也就是，然后还有就是经营的这些，就也像说的，眼界开了，就不在意别人怎么说的。嗯，感觉自己舒服才是最好的。嗯，所以我就也没那么在意一些东西了，像我爸妈说的，我就当没听。哎，不能说没听到，只是没有以前那么那么一句话。

说话人 1
就很烦躁那种。嗯，那你觉得我们这个团体对你最有帮助的一个地方。最有帮助的地方是什么。

说话人 2
呢？这有可能。

说话人 1
就像刚刚说的那个平提供了这样一个平台来帮助你。

说话人 2
最有帮助的吧。我觉得这场开我分不清楚罪，但是我觉得有非常有帮助的一个就是平台，一个就是那些知识嘛。嗯，因为这种大家一起交流知识肯定比你在网上骂我们目的的搜要靠谱很多。嗯，然后来的也有效，你可以直接把这个疑惑说出来让大家一起讨论嘛。在网上的话就七嘴八舌的，可能聊着聊着看，发着搜着，可能也不是在搜其中的东西了。

说话人 1
嗯，那你觉得我们这个团体最大的特点是什么？

说话人 2
就是大家都很包容，然后就可以你可以素，你可以不用素质的，你可以随时也不用，哈哈哈，你可以畅所欲言，说一些自己的心，然后大家也很热情的帮你去解决一些你想要得到问题答案的一些问题，这就还有就是，嗯，主要还有就是这个船服还是非常有条理性的，好像每次都好像针对我们东西有引导嘛？嗯，也不像大家真的是想说什么做什么，就是不是一个纯的聊天，还是有一些知识性的引导。嗯，让我们这一团妇也能够，嗯，有收获的也是有欢乐的也是有，也很和谐的一起进行下去。

说话人 1
那如果你要给类似的有减重需求的同学推荐我们这个团体，你会怎么说？

说话人 2
如果想又能够学到东西，又能够交到朋友，就来我们团府吧。

说话人 1
好，那好，我们就可以了。
