# Supplementary material for: Helpful factors of group cognitive behavioral therapy in overweight and obese college students
Source: Front Psychol. 2025 Sep 12;16:1585765. doi: 10.3389/fpsyg.2025.1585765 (PMC12463828; doi:10.3389/fpsyg.2025.1585765)
Supplement: Supplementary file 5 [file Supplementary_file_5.docx]

**鲁珉玉 4422**

*2024年7月18日 下午 12:19
14分钟 29秒*

**关键词**

团体 饮食 印象 看法 心态 知识 老师 朋友 成果 分享知识 生活实践

**文字记录**

说话人 1
那我们就开始，嗯，首先就是我们在，我们已经一共经济了 8 次，然后也是一个很长期的一个比较持续的一个时间。那你在我们团体中的一个整体感受是什么呢？

说话人 2
我觉得挺好的，就是在团体里面大家这个氛围很好。嗯，就是也会有比较积极主动的成员，然后带动我们这一个话题的推进，讨论一些方法，我觉得非常不错， very good。

说话人 1
嗯，那从第一周，然后一直到最后一次这么大的一个时间挂图，这中间你的感受有没有什么变化呢？

说话人 2
嗯，一开始的话就是可能因为不太熟，然后有一个认识同学的环节，我当时心里想，我说哇，有点尴尬，因为很多人又要记名字，我要是记不住旁边同学的名字，然后就稍微会有一点顾虑，但是随着那个时间推进，我感觉就是和大家越来越熟悉。嗯，哪怕有的同学可能不知道名字，就是什么信息也不了解，但是每一周感觉固定会见到大家就会有一种熟悉的感觉，有一种归属感。

说话人 1
那一共进行了 8 周，那你我们这个团体有没有哪些事件给你留下了比较深刻的印象？或者哪些场景？你觉得一想马上就想起来，你觉得很印象很深的。

说话人 2
嗯，就例如我刚刚说的那个，第一次大家那个认互相认识的环节，还有就是最后一次那个互赠礼物，嗯，还有那个最后有一次赵老师就是呼吁大家都站起来拉伸了，唉，对，那个做的比较多。对，就这几个有活动的环节会让我印象比较深刻。然后其次就是有的时候是赵老师在分享那个知识，就例如那个饮食摄入的那个量的那个地方，嗯，我印象比较深刻，还有当时说喝奶茶，喝果茶，那个大家知道糖分很高，大家一瞬间都很震惊的，那个时候印象很深刻。

说话人 1
那在这些世界里的具体感受和反应是什么呢？

说话人 2
第一次的话就是感受，刚刚也提到就对，有点尴尬，但是还是比较期待我觉得。为我后面参加这个团服，我说之后会有一些什么呢？就会比较期待、比较好奇，然后分享知识的时候就会感觉收获满满，就觉得很满足，就是感觉自己获得了很多东西。然后当时大家那个一起站起来运动的时候，我觉得挺高兴的，就是感觉大家都立刻投入到实践里面来这种感觉，然后最后一次真的很感动，非常有一都当时有一点伤感了，虽然可能表面上没有表现出来，但心里已经感动的不行。我回去和我的朋友说，我说哇，太感动了，发现这个朋友不太感动了，大概就是这样。

说话人 1
那你觉得这些事件对你有没有什么影响呢？对你自己。

说话人 2
影响有的，我觉得就是以后有可能我比较感兴趣的类似的团普活动，我可能就会更愿意参加，因为这一次的体验是会比较不错的。嗯，然后有的时候也是会在生活实践里面吃饭运动的时候，就有的时候会回想起团服里面的活动，嗯，然后我就会觉得这些活动留给我的回忆会促进我继续往前走这么一个感觉。

说话人 1
就潜移默化的影响自己。嗯，带入生活当中呢。嗯，那我们团体在给你在运动和饮食方面带给你一些变化呢。

说话人 2
饮食就是最明显的一点，就是我从原来吃的很多，而且吃的不够健康，就是可能高油高盐还是什么油炸类的食物，到现在我就会比较注重这个营养配比，而且吃的量也会显著减少。假如说原来可能吃个一大碗的饭，我现在可能只能吃原来的 1/ 4 到 1/ 3，嗯，就能吃饱了，我觉得就是对自己饮食量和健康的这个配比上有很大影响。我现在每餐不吃蔬叶蔬，蔬菜那种叶子菜我就会觉得很不舒服。嗯，然后运动的话我就会刻意的去增加运动。原来我是不运动，我说能不坐车，那还是尽量的坐一下吧。没关系的，坐个车没什么的，现在就是能走就走，我觉得哪怕走路也是一种运动，而且走多了还挺爽的，也不知道是什么什么感觉，就是我看你们。

说话人 1
每天都走挺多的。

说话人 2
对，你在群里面。对，就是没事的时候就会绕学校到处走一走什么就觉得反而运动对，我现在的负担没有那么重，就心理压力没有那么大，因为原来体育也不好，一跑步锻炼就感觉老是会有别人窃窃私语的议论自己运动，当然实际上是没有这回事，可能想太多了。嗯，但是现在我觉得就走路，大家走在看，在街上你走路也不会怎么样。嗯，就算你在田经常跑步，也不会有人怎么想你。所以我觉得就是心态上有很大改变，也会更积极主动去运动了。

说话人 1
那你自己原来的话是有情绪性近视有没有？

说话人 2
这个倒是没有，真没有。

说话人 1
那我在，我们在团，你进入我们团体之前，你自己肯定首先是有一个期待的。那我们现在已经结束了，你觉得你的这些期待有没有得到满足呢？

说话人 2
有，我觉得我捡了特别多。我现在一回顾起来这个数据，我说，哇，对，这个数据真的很惊人，而且是我们。

说话人 1
团体捡的最多的。

说话人 2
我觉得就真的很触动我，而且就是因为五一放假不是回去了吗？我家里人、身边人看到我都是感叹我这个变化，都是鼓励我，我觉得就是带给我的触动真的很难，我觉得我应该要坚持下去，不仅是为了外表上好看，也是为了更健康的一个生活，也是让以后自己生活质量能更好。

说话人 1
那你那有没有得到满足的或者遗憾之类的呢？

说话人 2
遗憾可能就是减的不够多，然后中对还不够多，但是因为中途心里还是会有几次比较延长这个促进作用。对，就会松懈，然后可能还是中途松懈了一点，要不然我觉得可能自己会做得更好，但是还是挺满意的了已经。

说话人 1
嗯，那在这个过程中，你我因为你是我们团体减的最多的，那你做了哪些剧情的努力来帮助你来进行一个健康的。

说话人 2
减重？嗯，就是控制饮食量，说实在话就是这一点，因为我团体绝大部分就是前程到中后期，其实基本上没有刻意的去增加运动量，嗯，就是也是一每天就 1 万多部， 9, 000 多部，这种就是克制饮食量。只吃一个拳头的碳水和两个拳头的蔬菜和一个拳头的蛋白质，就是严格这么吃。

说话人 2
嗯，而且包括像赵老师说那种带有汤汤水水的泛指当时是一口不吃，就是当时一开始吃可能会不习惯，就会觉得好，是不是有点饿？嗯，但是实际上是心里觉得自己饿，肉体上其实是已经满足了的，然后保持了比较长的一段时间，吃了几周以后，我会发现自己心理上也不觉得饿，而且就是有的时候可能没有吃到刚刚说的这几个量，嗯，我就会觉得自己已经饱了，就已经不是很想再吃了，特别是那个进食顺序也会有变化。

说话人 2
原来可能就是先吃肉，然后加着饭一起吃，现在就是会先吃蔬菜吃多的时候，因为吃蔬菜肯定是要嚼的，咀嚼的这个过程中就可能嘴巴上就得到了一点满足，然后吃进去之后再慢慢的去吃一些，假如说实在觉得自己很饿，再吃一点饭，然后再吃一点肉之类的，然后就这么一个顺序，嗯，然后会把进食时间稍微拉长一点啊。

说话人 2
我之前也说过说，嗯，原来吃饭非常快，可能 10 多分钟就全部一口气吃完了，现在就是会啊，假如说一边刷视频一边吃饭，嗯，或者是一边打字一边吃饭，虽然饭可能会凉，但是我觉得如果进食的时间拉长了，对自己这个胃里面这个满饱腹感的一个体验就会比较深刻。假如说我吃了 10 分钟左右，我这个时候就会停下来，我去觉察一下自己现在是几分饱，我说大概吃了哪些东西？我现在还饿不饿？我就会有意识的去想这个部分。嗯嗯，然后差不多饮食就是这些，然后运动的话就是会鼓励自己就多走一走，虽然我还是很不喜欢跑步之类的运动，比较伤害什么膝盖，这个是真的有一点，但是就会多走一走，然后走路的时候一边会想说这也是一种消耗，然后不知不觉就会走很多，就是走起来会就走上瘾了有一点。

说话人 1
那你觉得自己现在的状态怎么样？你自己会怎么评价？我觉得。

说话人 2
现在如果是五点几分的话，哈哈哈，那差不多就是 4 分左右，感觉就是现在的就是无论是肉体上的这个感受，还是精神上这个感受都是比较好的，就有一种很好的期待往前走的这么一个感觉。

说话人 1
但看法，你对自己的看法有改变。

说话人 2
吗？看法改变的话我觉得会觉得自己还是挺舍得努力和付出的吧。原来可能就会觉得你怎么不行，对自己可能会比较多的有否定的这么一个倾向，但是我觉得特别是现在这个很显着的成果摆在我的面前，我会觉得其实鲁豫你做的真是挺不错的，而且就是从自己和他人身上都得到了积极反馈，我觉得对自己的看法有改观，我说我是可以做得很好的，我是有这个能力的，我不会再一味的否定自己，你不行，你不行，我会鼓励自己。我说你其实做得很不错，你可以坚持下去，你一定会做得更好的，有可能对自己看法更积极正向。

说话人 1
那你就是你发生了这么多改变，那你觉得这些变化对你的生活有没有什么影响？

说话人 2
嗯，有吧，就虽然我身边的大学同学可能没有太关注到什么我的外形变化，但是我觉得我的心态改变了之后，我跟他们的交往有一种潜移默化的改变，就是我不会因为别人一点点反弹，或者是就是很普通的行为，我会开始很敏感，怀疑自己是不是做错什么，我会觉得这样就这样呗，人家也没有明说，我也不会多去想这一方面。所以我就是觉得自己可能不太过于在意外，外界的那种评价，也不会太敏感的对于别人审视自己的目光这种太在意就是感觉更能做自己，就是自己想做什么做什么了。

说话人 1
嗯，就是主要是心态上的一个变化。嗯，那你觉得有哪些因素促进了你的这些成长会变化呢？

说话人 2
嗯，首先就是自己对自己的一个认识，就是我可能不再是过于的把主观的情绪和想法强加在自己的认识上。我说你不行，你这里不好，那里不好，你太敏感了，什么什么的。我会更客观的去分析自己的一个心态，假如说我现在心情不好，我会去思考，我说我是为什么心情不好，这一点是真的吗？还是你自己想太多了，然后会更客观的去看自己，然后还有啥来着？问题是。

说话人 1
哪些因素嫌动你？

说话人 2
动力的话可能还是想变得更好，因为其实从小到大都是一直比较胖，然后也不是太注重打扮，但是我有这个，上了大学，我想我说哇，你人生青春就十几岁，二十几岁，就这么一次，我说不可能一辈子都顶着这副躯体去吧？我说还是有机会想让自己变得更好。嗯，更健康也是希望就是。什么身边的父母不太操心，和身边的朋友可以打交道，更来这种情况就可能还是有外界的因素促使我改变。

说话人 1
那我在我们团体中有没有什么因素促使了你的成长和。

说话人 2
变化？嗯，有吧？就是像那个杨平学姐，她那个比较积极的参与到这个活动里面就会有反馈，包括我们在群里面有的时候也会就自己发的一些图片，有一些聊天，我会觉得这个团体不只是我一个人在参与，就是有成员之间的互动。嗯，我会觉得更有投入感，就是不止我一个人在努力，我觉得这种促进作用是很重要的。

说话人 1
那你觉得我们团体对你最有帮助的地方是什么呢？

说话人 2
嗯，最有帮助的地方可能就还是行为习惯上的改变，就是我会知道一些更科学的原理，然后自己去实践，发现真的很有用，我会就这样坚持下去。我觉得就是这种引导的作用会很最重要，就是引导自己去往一个方向走的这种指引作用，要不然就有点像梧桐苍蝇一样在那里乱转，是很耗费精力。

说话人 1
的。网上有很多，但是还是不一样。对。

说话人 2
就是感觉网上教授的知识和现场轻声有人告诉你，有人传授你和大家有实践的这么一个过程，我觉得是很不一样的。

说话人 1
那你觉得我们团体最大的特点是什么？

说话人 2
特点就是感觉大家虽然好像不是很熟悉，但是在心灵上已经打通在一起了，就是这种感觉，好像我们就有点像现实版的那种网络聊天室，虽然我们好像彼此之间不是很熟悉，但是可以通过别人的举止言行，大家会有一个共同的目标，还是觉得心是在一块的，就是这么一个感觉。

说话人 1
那如果你会给类似就是有减重需求的同学推荐我们团体，你会怎么说呢？

说话人 2
我会说，我说哇，包管用的，你尽管来吧。包管用的只要你投入进来，一定会有成报成果和回报的。我说包管用，你相信我，你看看我，我就会开始列举自己的这么一个经历。

说话人 1
好，谢谢。
