# Supplementary material for: Helpful factors of group cognitive behavioral therapy in overweight and obese college students
Source: Front Psychol. 2025 Sep 12;16:1585765. doi: 10.3389/fpsyg.2025.1585765 (PMC12463828; doi:10.3389/fpsyg.2025.1585765)
Supplement: Supplementary file 6 [file Supplementary_file_6.docx]

**隆宇通 1824**

*2024年7月17日 下午 10:54
6分钟 42秒*

**关键词**

团体 焦虑 饮食 信心 观念 氛围 情绪 心态 整体氛围

**文字记录**

说话人 1
你分享一下你在我们团体中的一个整体感受和体验。

说话人 2
就是在我们的团体中我感觉大家其实相处都很融洽。然后我也感受到了，就是同为减肥人的那种信心，但是可能我是一个比较内向，然后，嗯，比较懒的人，然后可能大家的建议就是很少回复，注意实践这种的人，但是整体氛围感觉还是非常好。

说话人 1
的，就是觉得挺轻松愉悦的氛围。是但随着我们，因为我们一共有 8 次，然后随着这个时间的推移，你的一个心态感受经历了怎样的变化呢？

说话人 2
就是刚开始的时候，刚参加团服的时候我还是很有信心的，但是进行到后面的时候，刚开始实行的一些那种措施，我是可以进行下去的，但是到后面我可能就坚持不下去了，可能就会有点厌烦。然后再到最后的话就是放平了，就是没关系，就是感觉还是从最初的那种兴奋，然后到中间的有点厌烦，然后再到最后的就是比较放松的。

说话人 1
最后就是比较平和的一种心态哈。那你在我们团体中有哪些事件或者时刻给你留下了深刻的印象呢？

说话人 2
嗯，事件就是当时有个同学跟我说他靠不吃零食，然后也没有特别运动，但是几周下来他就减了差不多 10 斤，这个是给我非常大的震撼，然后也给我一点信心的那种。

说话人 1
那这个事情对你有什么影响？

说话人 2
就是让我清楚的就是比较了解到就是饮食方面的影响还是非常大的。然后就是对减重这件事情。

说话人 1
嗯，那因为我们那个团服也经历了这么多次，嗯，那我们也分享一些进食啊，运动方面的知识。那你自己有没有在就是这个团购中，就是。嗯，改变你生活的一些运动或者饮食方面的东西。

说话人 2
嗯，饮食方面的话就是我会对重油、重盐这一类的食物稍微注意一点，少摄入一点，嗯，能不摄入就不摄入这种。然后运动的话，嗯，可能比较忙，然后就也没怎么应的。然后，嗯，但是稍微也会多走点路，少坐那个小白车了。

说话人 1
就是还是会有那么一点的变化，是吧？

说话人 2
对，就是会稍微注意一点。

说话人 1
那这些变化对你有一这些这改变就是你的饮食运动改变对你有什么影响？

说话人 2
没有很大影响，因为改变异这些都是比较小的方面，就还是能做到、能坚持的。

说话人 1
那你就是你有没有过情绪性近视的行为？

说话人 2
还是有过的。那。

说话人 1
你参加这个团腐之后有什么新的变化或者感受？

说话人 2
就是感受就是情，就是有情绪的时候不会就是盲目的就是说自己跑下去买点东西吃啊一下子。嗯，就是也会，就是换一种方法去疏解那种情绪，就比如跟同学聊天那种，或者给爸妈打电话的那种。

说话人 1
那你进入我们团府之后，你在那个进入前的一些期待有没有得到满足？

说话人 2
进入前的期待，期待的话其实当时我不是抱有很大的期待，我就是想要尝试一下的。但是进来之后确实也学到了挺多东西的。

说话人 1
嗯嗯，那在我们这个团伙过程中，你自己有没有付出哪些努力来帮助自己实现自己的减重目标呢？

说话人 2
嗯，好像比较少，因为我是一个那种真的是说有点懒的，躺在寝室就不想动的那种人就是。嗯，努力不到，不多吧。

说话人 1
那你如何评价你现在的一个状态呢？

说话人 2
我感觉现在这个状态还挺放松的，就是快期末考试了，还是有点烦的。

说话人 1
嗯，就是你现在就是参加我们团腐之后的一个状态，就是比如说信心或者是你的焦虑性这些有没有什么变化？

说话人 2
如果是对体重方面的焦虑性的话还好，因为减轻了很多，因为发现其实，嗯，减下来是一个很好的东西，但是不减下来的话其实也还好，因为发现大家其实对于肥胖这件事情的认知就是没有那么严重，也不像就是网上说的那种，那么就是制造那种室内焦虑就是不好，那种什么，嗯，只要保持一种健康的生活方式就是可以的了。

说话人 1
那你觉得我们这个团伙对你最有帮助的地方在哪呢？

说话人 2
最有帮助的地方应该是，嗯，改变了我的一种观念吧。嗯，因为之前就总觉得瘦下来是最好。嗯，就是那种，嗯必须要符合大众的审美，然后嗯，白又兽这种审美这种类型，但是到后面的时候就是大家通过大家的聊天方法，就是大家对于肥胖这件事情其实没有想象中那么焦虑的，因为这种同类人之间的那种交流就会让你感觉到非常的舒适，然后我也不会给你就是造成太多的烦恼那种。嗯，反正心智平和了很多。

说话人 1
嗯，你觉得我们这个团服最大的特点是什么？

说话人 2
最大的特点就是这种群体性的讨论，然后和那种，嗯，观念上的事实就是行为上的那种，嗯，怎么说？改变吧。

说话人 1
嗯，那你在我们这个团服当中有什么你觉得遗憾的地方。

说话人 2
吗？遗憾的就是嗯，跟大家之间的关系，嗯，少参加了几次，然后跟大家之间的关系就是没有到达，就是那种志同道合的程度。

说话人 1
就是还没有更深。

说话人 2
硬，就更深入一点大跟大家。那。

说话人 1
如果你要给身边有类似的减重需求的同学推荐这个团体，你会怎么说呢？

说话人 2
我就说，嗯，这个团体它是从观念上去改变你的那种减肥诶方式，就是你可以去尝试，然后可以加入我们这个团体，是一个非常温暖的大家庭。

说话人 1
好，那就这些，嗯。
