# Supplementary material for: Helpful factors of group cognitive behavioral therapy in overweight and obese college students
Source: Front Psychol. 2025 Sep 12;16:1585765. doi: 10.3389/fpsyg.2025.1585765 (PMC12463828; doi:10.3389/fpsyg.2025.1585765)
Supplement: Supplementary file 7 [file Supplementary_file_7.docx]

**李岩 1913**

*2024年7月17日 下午 10:54
6分钟 53秒*

**关键词**

团体 焦虑 收获 体重 分享 知识 朋友 饮食运动 整体状态

**文字记录**

说话人 1
我们开始了，是你分享一下你在我们这个团体中的一个整体感受和体验。

说话人 2
我整体的感受就是因为之前在减肥的路上都是自己一个人，然后就是突然找到了一个集体，大家可以在这个团体中分享自己的问题，或者就是或者自己的进步什么的，我就觉得是一个可以共同进步这种感觉。

说话人 1
嗯，就是比较轻松快乐的。对，嗯，那随着我们这个时间的推移，因为我们一共有 8 周嘛。那你的一个感受有经历怎样的变化呢？

说话人 2
就是最开始的话就是让我们做一些，最开始就是属于那种要求，也算是，然后就是感觉是一种要求，但是到了后来慢慢的就是已经，就是昨天说的已经成为一种习惯了，就不会说。是啊，我每天想着就是被要求的感觉和自己愿意去做的感觉是不一样。

说话人 1
的，就是刚开始有被迫，后面慢慢的就是有主动的那种感觉了。对，那你在我们团体当中就是有哪些事件或者时刻给你留下了深刻的印象吗？

说话人 2
其实我觉得整个团体对我来说都是印象很深刻，特别是在每次讨论的时候，每次在不同的同学给出不同的意见什么的时候，我觉得每一次都很值得被记住。

说话人 1
那大家在讨论的时候你有什么感受呢？

说话人 2
我的感受就是哇，我又收获了好多新知识啊。那我又该怎样？嗯，就是更多的就是就学习，抱学习的态度更多是。

说话人 1
那就是我们在这些团妇当中的一些讨论，这些事情对你自己有什么影响呢？

说话人 2
影响那就多了，哈哈哈，那学到很多，然后就会也会说，嗯，照着做吧。嗯，就差不多，就是学更多学习付出实践。

说话人 1
对，那，因为我们团体也有 8 次，这八次我们有讲到很多就是饮食运动方面的知识跟一些技巧。那你对它，对你带来了就是怎样的变化吗？

说话人 2
当我真正就是按照我们所学习的来做下来的话，其实对我自己的改变还是蛮大的，就是从，不管是从身体还是从心理上面，我就从发自内心的，我觉得我该这么做，然后这体重自然而然也就减下来了。

说话人 1
所以你是有，比如说你是有，就是放在生活中，就是你运动也有增加，是吧？你平时也运动？嗯，对啊，那你就是在团辅前，团辅后运动的频率有变化吗？

说话人 2
嗯，那这个的话其实还是有一点，就比如我前两天不是天气不太好嘛？嗯，然后平时这种时候就可能在宿舍呆着，但是我想着不行，我得去动一动，我就去泡个健身房。

说话人 1
那这些变化它对你有什么影响吗？就是团体当中一些饮食运动的改变对你有什么。

说话人 2
影响？就是首先来，至少体重我们下降了，对吧？然后就让我就不管，其实我觉得不管体重有没有下降，但我在这个过程中我收获了自信，我也收获了自律。

说话人 1
就是你在我，嗯，进入我们团体之前的一些期待就是得到满足了吗？

说话人 2
就是如果一定要算的话，可能还是差了一点点。嗯嗯，差几斤？

说话人 1
就是有满足，只是没有满足程度，没有达到百分百，是吗？嗯，对，好，那就是你自己做哪些？就是你付出了什么努力来帮助你自己实现的一个减重目标。

说话人 2
自己做的那就是控制自己想吃的欲望，到后来慢慢就不想吃了，然后就是在不想动的时候，还是就是让自己去，还是要动起来，就不会就是坐着那种。

说话人 1
那你现在如何评价你现在一个整体状态呢？

说话人 2
我觉得现在的状态就是我，我觉得我现在的状态是一个非常好的状态，因为我其实也不怎么吃，不是说不怎么吃，就是没有之前那么就是爱吃的，嗯，然后我运动也很规律那种，嗯，然后我就算不掉，我觉得嗯比之前也会好很多。就要如果我在运动的话，体肯定体型会更越来越好的那种。

说话人 1
那你现在就是和焦虑这些有什么变化。

说话人 2
吗？焦虑的话其实之前更多的焦虑就是。嗯，我自己会想。嗯，就是好，就瘦一点，然后穿一些好看的衣服，怎么样？然后再加上家里人就是总是在说你胖了，你该减肥了。那现在我慢慢就是至少数字也在下降，然后体积量慢慢变好，我就觉得就可以不用想那么多了。

说话人 1
就是焦虑程度下降了。嗯，对，那你认为有哪些因素它促进了你在团体中的一些成长和变化？

说话人 2
哪些因素我觉得首先还是最重要的，还得是团体的力量。嗯，如果一个人的话肯定是不太现实，然后再加上就是，嗯，自己也要自律才行。

说话人 1
好，那你觉得我们这个团体它对你最有帮助的地方是什么？

说话人 2
最有帮助的地方？怎么说呢？就是至少有一群志同道合的朋友，然后再，嗯，更多自信的嘛。

说话人 1
你觉得我们这个团体最大的一个特点是什么？

说话人 2
最大一个特点。

说话人 1
没关系，没关系。就是那我们团体有哪些地方你觉得做得不好的可以改善的吗？

说话人 2
做得不好的吗？就是我个人属于那种，就是，嗯，不太发言的那种，就是，然后如果说是，就是肯定就是相互讨论，我一定要愿意的话可以自己讲，但是如果轮着来，当然肯定是为了了解每个同学，都是，有时候可能真的不是很想讲。

说话人 1
好好好，那如果说你身边有类似有减重需求的同学，你会怎么给他推荐我们这个团服？

说话人 2
我就是一个很好的例子，就是你。

说话人 1
以自身为履历的一个达到的。

说话人 2
目标。对啊对啊，就我在不是那个表班题中的表，我拍给我朋友看，他说请教教我，谢谢。

说话人 1
哈哈哈，好，那我们今天那个访谈就到这了。好，谢谢你，谢，拜。拜拜。
