# Supplementary material for: Helpful factors of group cognitive behavioral therapy in overweight and obese college students
Source: Front Psychol. 2025 Sep 12;16:1585765. doi: 10.3389/fpsyg.2025.1585765 (PMC12463828; doi:10.3389/fpsyg.2025.1585765)
Supplement: Supplementary file 8 [file Supplementary_file_8.docx]

**雷小鹏 1836**

*2024年7月17日 下午 10:54
6分钟 55秒*

**关键词**

知识 焦虑 团体 认知 技巧 分享 印象 收获 运动饮食 运动技巧

**文字记录**

说话人 1
那我们现在开始就是你分享一下你在我们这个团组当中的一个整体感受，这个体验。

说话人 2
就是感觉这是团服中收获了很多那些专业的知识，还有的话就感觉互相交流的话氛围非常的轻松快乐。嗯，我还有一个就是感觉也是一种很大的一起志同道合的朋友，感觉挺有福利的。

说话人 1
那就是我们这个团服因为有 8 周，那随着这个时间的推移，你的一个感受经历了怎样的变化呢？

说话人 2
如果刚开始的话就是兴致非常的高，就是有努力的在那里学习，然后就是减肥的动力，非常热情，非常之高。然后后面就是慢慢的间隔，时间比较长，之后就是比较顺延的话，就是让人感觉就动力有点有所下降，然后参加一次的话就是从轻上高一点点，然后就这样子往复。

说话人 1
那你在我们团体中哪些事件给你留下了深刻的印象呢？

说话人 2
就是互相交流着，比如老师科普一些专业知识有推荐，比如说那些好说回应卡之类的。

说话人 1
嗯，那你对我们就是这些给你留下深刻印象的这些事，你的感受是什么呢？

说话人 2
感受就是感觉一种收获的感觉，就是收获的那些知识，还有那些获得了几鼓励？嗯，懂得。有些那些想吃的东西，比如那些近视之类的。嗯，就是感觉就是很平常的。

说话人 1
那这些就是你感兴趣的留下深刻印象的事情，对你有什么影响？

说话人 2
因此就是我可以更加注重饮食方面的那些类型，还有进食的量，还有的话就是运动的 in the love 那些技巧。

说话人 1
那经过我们这 8 次的团体，那你在日常生活中的运动方面和近视行为上有发生怎样的变化吗。

说话人 2
近视方面的话就是就会改变那种尺色特别油腻的东西，比如说之前也说过那个花菜比较有益，然后就是尽量的多吃了一些碗芹菜那边的方面，烤肉的话运动的话就是也获得很多运动技巧了，运动能量也有所提高的话也有所提高。

说话人 1
就是食物上可能就是更懂得如何搭配了。

说话人 2
对，就是。

说话人 1
更，然后运动的频率也有提高。嗯，那你对于你运动饮食方面的一些变化，这些变化对你有什么影响吗？

说话人 2
最直观的影响的话就是体重在慢慢的下降。

说话人 1
那你有没有情绪性近视啊？情绪性有过这种行为吗？有。那你来我们这个团体里有变化吗？

说话人 2
就有时候以前可能话就是想着无所谓的话，就是经过什么事情的话，一般比如说上课，上了很多课，一直很多课的话就是很累，很累的话就会多吃嘛。那有时候每次早上的话可能。嗯，那种也会多吃，就是参加团腐之后就会知道不应该去那些，因为一些特别的事件去增加自己的近视。

说话人 1
就更理性了，是吗？嗯，那就我们这个团辅结束了，你就是你之前进来的一些期待得到了满足吗？

说话人 2
还是得到满足？还是慢慢有所体重有所下降？嗯，也学到了很多减肥方面的知识。

说话人 1
那你在我们这个团伙过程当中，你自己有做了哪些努力来帮助自己实现这样一个减重的目标呢？

说话人 2
嗯的话就是注更注意重搭配那种饮食的，还有吃饭速度也有所下降，然后还有增加了运动量有所增加。嗯，运动的。

说话人 1
方法，那在我们这个团辅过程中你自身发生了什么改变？什么？自身感觉，自身就是你整体有了什么样的变化？

说话人 2
是感觉就是自身的话就是懂得更多技巧能够。嗯，听说就是你的体型也有所，体型也变得更加的是没有比之前那么肥胖，然后就是。

说话人 1
就减重，知识上有变化，然后外形也有变化，是吧？对。

说话人 2
还有心态上有变化，还有对待这种肥胖子心态也有所变化。

说话人 1
嗯，那你如何评价自己现在的这样一个状态的话？状态他可能是积极、消极。

说话人 2
或者就是身体可能因为减重更健康了，然后对减肥的那种信心也有所增长。嗯，就是更加有信心能够减下去。那。

说话人 1
你那继续对这方面的焦虑程度。

说话人 2
焦虑程度也肯定是有所下降的。以前因为各种原因讲 difficult one 就是有点会焦虑，但是现在就说没有那么焦虑了。

说话人 1
嗯，那你觉得是有哪些因素造成你现在的一样的变化呢？

说话人 2
可能就是团府的那些成员都互相交流知识的班，还有团府一起交流那些东西，然后给了一些动力吧。每次常见玩唐夫，这是。

说话人 1
团体的力量。嗯，那你觉得我们这个团服对你最有帮助的地方是什么？

说话人 2
就是学到了很多心理，从心理改变的认知的方法，然后就是达到减重之前学到那些知识，正念近视、法难之类的。

说话人 1
你在这个团赋当中有没有什么遗憾？

说话人 2
以后就是有几，有一两次可能没来完，因为有事没来，没有学到知识。

说话人 1
你觉得我们团府最大的一个特点是什么？

说话人 2
就是一些改变认，从通过改变认知的方法去减肥，而不是去那些。

说话人 1
而不是直接通过外力。

说话人 2
对外力，而是通过改成内心中的那些认知来减肥。

说话人 1
嗯，那你觉得我们这个团妇有没有哪里做得不好的地方需要改善呢？

说话人 2
嗯，就是。

说话人 1
没有，哈哈，这么完美。好，那如果说你要给类似有减重需求的同学推荐我们这个团服，你会怎么推荐呢？

说话人 2
但是讲我经过几次团服之后减重的成功案例给他进行分享。嗯，就。

说话人 1
说你自己在这里面确实得到了减重，是吧？对，达到目标。嗯，好，那基本上就是这些，谢谢你，结束了。
