# Supplementary material for: Helpful factors of group cognitive behavioral therapy in overweight and obese college students
Source: Front Psychol. 2025 Sep 12;16:1585765. doi: 10.3389/fpsyg.2025.1585765 (PMC12463828; doi:10.3389/fpsyg.2025.1585765)
Supplement: Supplementary file 9 [file Supplementary_file_9.docx]

**扈俊欣 2988**

*2024年7月17日 下午 10:55
10分钟 51秒*

**关键词**

焦虑 团体 食物 印象 观念 活动 整体状态

**文字记录**

说话人 1
那我们现在开始，嗯，你分享一下你在我们这个团体中的一个整体感受和体验，就是经过我们巴西的这样一个团。

说话人 2
整体的感受就是挺轻松的，就是大家像朋友一样什么都说，然后也嗯，听别人他的经历，或者是把自己的经历分享出来，然后也得到了很多方法，得到了很多支持，而且就是在嗯通过平时打卡的时候也有这种相互的鼓励。

说话人 1
那我们就是因为有八次，然后随着这个八次他时间的推移，你的感受经历了怎样的变化？

说话人 2
感受吗？刚开始的时候因为，嗯，第一次参加这张这种团腐吗？第一次结束团腐这种活动形式，刚开始觉得特别新奇。嗯，前几次的时候因为每周一次也是挺。嗯，准时的，就是按期来开展，就是对我团腐之后的平时的行动影响还挺大的，但是后来可能因为也有一些延期的情况，也有后来忙起来了就会影响的，没有这么多。

说话人 1
去感受感受金额。

说话人 2
在感受吗？嗯，感受应该是什么方面？自我就是。

说话人 1
比如说我现在感到很轻松愉悦，我现在感到很焦虑。

说话人 2
最开始是感觉新奇，然后后面就是来的时候可能有点追不安的，因为这两周表现可能我自己觉得不是很好，没有按照团普预期的方向发展，就是来的时候还挺有点不是很情愿来，但是后来在团普过程中就会被就是把这种不好的情绪消解掉了，就是觉得被包容，觉得就很自在。嗯，最后就比较自洽了。后面，嗯，对对，是的。

说话人 1
那你在这个团体当中有哪些事件给你留下了深刻的印象？

说话人 2
嗯，就是第一次尝试那种正念进食的时候，印象特别深刻，感觉从来没有那么那么认真的去感受过他。

说话人 1
那你当时就是正面进食的时候，你的具体的感受是什么呢？

说话人 2
我具体是感，就是我感觉我整个人都特别宁静，我的注意力就放在这个东西跟我自己身上，嗯，特别轻松。

说话人 1
嗯嗯，那就是这个正面进食它对你有什么影响？

说话人 2
就是改变了我对食物的一个态度，就是我有想吃的东西，这不要紧，没什么，我可以，嗯，用它来做个调剂，让我享受这个过程，而不是要怎么追求什么量的这种。

说话人 1
那就是，嗯，因为我们这个团体就是也是减重方面的嘛。嗯，那你经过我们这 8 次团体，那你日常在运动、进食行为上有什么变化？

说话人 2
运动上面就是，嗯，平时就算，嗯，就是正常的上课，放上课之就是，嗯，可能平时日程排得比较满，但是会见缝插针的去多走两步，能会刻意的多做一点点运动，可能应该要把它把多走这些路应该要散到，就是我有意的去增加这个运动量里面。然后进食的话就是一个是对食物，就是能跟食物友好的相处，不会说刻意的去抵制，也不会说，嗯，就是也不会那种很病态的渴望那样，嗯嗯，对的。

说话人 1
那你就是你的这两方面的一些变化就是对你有什么影响吗？

说话人 2
嗯，让我变得更健康了，还有就是因为在团普里学到这些方法，也有不自觉的就落实到了生活里，然后我每天久坐之后可能起来活动一下，然后拍一拍。对，我就让我感觉我的状态要好一些了。

说话人 1
嗯嗯，那你知不知道情绪性近视是什么？

说话人 2
嗯，就是我焦虑不安或者难过痛苦的时候，就是通过进食的这种来改，就是从里面获得一点。

说话人 1
那你有这方面的一些行为。

说话人 2
吗？有，就是比较焦虑的时候，之前可能会尝试，就是之前可能会有这种情况，焦虑的时候可能会说你这件事情我特别特别不想干，可能会先去吃个饭。怎么样？吃点东西。

说话人 1
那你参加这个团府的话，你这方面有什么新的变化或者感受？

说话人 2
嗯，有的就是团伙过程中有提到说什么从食物里面获得安全感是天性，就是后来就是比较自洽，但是后来就是慢慢的可能通过其他的方式来获得安全感觉哦。

说话人 1
嗯，那我们这个团体现在结束了，你就是进入我们这个团体的期待是否得到了满足？

说话人 2
那你期待吗？嗯，就是我期待还挺高的，但是因为我就是过程中只是我觉得，我觉得我参加这个团妇如果把他，嗯每次打卡都做的特别好，这种投入是一种，然后得到相应的回报就可能也比较高，然后我只是坚勉强勉强参加了，结束下来了，跟着结束下来没有不能放弃，这应该是另一种，所以。嗯，我最开始预期是，预期的是我投入的高，得到的回报也高，现在就是投入的也少。但是我对这个结果其实挺满意的。

说话人 1
就是你对于你付出的一些东西得到这个结果其实是挺是的。嗯，那你在团服过程当中你做哪些努力来实现自己的减重目标吗？自己有哪方面的？嗯，付出。

说话人 2
嗯，就是。嗯，去改变我日常的习惯，会早睡会，饭前喝水，然后会多增加一点运动这些。嗯，就是把一一，就是我知道那个习惯他出什么地方出了问题，我会把这个问题慢慢的解决。他。

说话人 1
那你在我们这个团伙当中你自己有什么样的改变？就是整体的有什么样的改变，然后你对自己的这个改变有什么样的？嗯，看法。

说话人 2
的改变就是我好像跟自己合集的比较多，就是我之前可能有点不好意思，觉得就是有一种什么一种，嗯，不好意思打扮的感觉，因为体重这个事情确实有这种焦虑。然后来就跟也是跟团伙的小伙伴们一起，就是看他们都做的挺好的，可以想穿什么其实也没有很大的顾虑就。嗯，能够正视这些。噢，就是对自己生态没有这么焦虑了应该。

说话人 1
那你如何评价你自己现在的一个整体状态？

说话人 2
我觉得我现在的状态要哪方面的呢？就是你。

说话人 1
对自己，你觉得你现在是更积极的面对生活，还是说比较负面或者什么什么的焦虑情况啊？这些。

说话人 2
我觉得还挺记得，因为，嗯，就是像谈服务当中说到的就是掌握。嗯，先控制自己的体重，也是对自己生活的控制，就是我在有意的去做这些方面的时候，同时也这些反馈给了我一点自信，就是让我就算期末周很忙也不会很崩溃。然后我感觉有些事情是在按我的控制，这个在往这个方向发展的。

说话人 1
嗯，所以你现在就是也不会很焦虑。对的，没有。

说话人 2
那么焦虑了。

说话人 1
那你的，你认为你现在这些变化是哪些因素促成的？

说话人 2
这些变化就是整个团服过程中也不能说具体哪一次，但是每一次都可能会有这么一两个点，就是触动到我，然后让我觉得，嗯，就是一种观念，就是改变了我的想法，然后就会一点一点积累到现在，导致我自己身上几就是跟嗯怎么说呢？噢，可能是我从其他的小伙伴身上学到的东西，然后就把它变成了我自己在做的东西了。

说话人 1
就团体的力量，对，团队，那这些你的变化对你的生活有什么影响？

说话人 2
对我的生活的影响。

说话人 2
让我变得就是这个团服，让我对我自己，嗯，更包容了，让我变得更自信了，然后就影响到我的日常和其他老师和同学的交际。

说话人 1
那你觉得这个团体对你最有帮助的地方在哪里？

说话人 2
嗯，最有帮助的地方就是。嗯，那些我习以为常的观念就是他们现在已经没有这么根深蒂固，就是有受到了改变吧。

说话人 1
那你在这，我们这个团体当中有没有什么遗憾呢？

说话人 2
遗憾吗？遗憾就是。嗯，后半学期其实还挺忙的，如果是前半学期就好了。说不定会投入的更多，然后也会收获更多。

说话人 1
就是自己投入上可能有些。

说话人 2
对的，有点。

说话人 1
嗯，那你觉得我们团府最大的一个特点是什么？

说话人 2
它不特点就是。嗯，每个人都有，都想说什么就说什么，就是大家都会很包容的去听，然后自己发言的时候也没有那种，嗯，担心我说错了，或者说的不怎么样，这样的。

说话人 1
那如果你要给类似的有减重需求的同学推荐我们这个团服，你会怎么给他推荐？

说话人 2
嗯，我想一想给类似的有几种汽车，这应该怎么推荐呀？用。嗯，去的肯定不亏。

说话人 1
就是他大概我们团富在你心中是怎么样的，你就会怎么推荐。

说话人 2
嗯嗯，我觉得就是。其实。嗯，就是你一周里其实特别忙碌，然后这些乱七八糟事情让你感到很疲惫，然后你每周拿出这么一个半小时来参加团服，其实是给你的心灵，就是尤其到一个放松的东西，甚至一个半小时你可以什么都不想，把手机也放得远一点。然后我们就专注在这些交谈里，因为平时很少有机会把，就是大家坐下来把这些事情开诚布公的谈一下。

说话人 1
这是一个疗愈的作用，是吗？对的。嗯，好，那基本上就这些，好的，可以了。

说话人 2
谢谢。好，不客气，拜拜。
