# Supplementary material for: Helpful factors of group cognitive behavioral therapy in overweight and obese college students
Source: Front Psychol. 2025 Sep 12;16:1585765. doi: 10.3389/fpsyg.2025.1585765 (PMC12463828; doi:10.3389/fpsyg.2025.1585765)
Supplement: Supplementary file 10 [file Supplementary_file_10.docx]

**陈克壁 2453**

*2024年7月17日 下午 10:54
10分钟 10秒*

**关键词**

团体 焦虑 饮食 身体 动力 频率 身材 体重 饮食运动

**文字记录**

说话人 1
好好好，那我再来一次，去分享一下你在团体中的整体感受和体验。

说话人 2
我觉得在这个团体中整体的氛围是比较轻松愉悦的，就是大家共同探讨就是减肥的一些方法，还有一些生活习惯，这些还是感觉很快乐的。

说话人 1
那你就是因为随着这个时间的推移，我们的 8 周，那你前期、中期、后期的一个，嗯，感受它有没有一个变化或者一个起伏？

说话人 2
前期的话因为，嗯跟他们也不是很熟，就是大家都比较陌生，然后多多少少会有点害羞，然后不敢表达自己的想法。嗯中期的话就是稍微有一点熟悉了。嗯，我觉得就是更敢袒露一点自己的心扉了。后期的话我感觉，嗯，就是大家已经有了明确的那种认识，然后我觉得大家聊天的那种氛围就明显有所不同。

说话人 1
那就是在我们这个团体当中有哪件事给你留下了深刻的印象，或者哪个场景？

说话人 2
我觉得是那个分吃东西享受就是。嗯，正念。

说话人 1
那次。是吗？正念进食。

说话人 2
吗？对对对，正面进食，因为让我深刻体会到。嗯，就是。嗯，吃东西就是。嗯，小时候它就没有之前那么狼吞虎咽了。

说话人 1
那你针对这个就是吃，就是这一次的正念这个进食你的具体的一个感受是什么呢？你。

说话人 2
当时我记得好像分给我的是葡萄干。嗯，然后我当时放进嘴里的时候就是品尝到葡萄干的那种甘甜味，然后他品尝完之后，他感觉就是在口中还是有余香的，就是那种回味无穷的那种甜，然后给我感觉很好。

说话人 1
就所以当时就是感受挺好的，是吧？嗯，那这个正念进食这件事情对你有什么影响？

说话人 2
嗯，我觉得这样，这个对于我进食的话，我可能在生活中吃东西的话会比较慢一点的，就没有像之前一样狼吞虎咽了。然后吃东西的话慢一点有助于我消化吸收，其实也挺便益于我减肥的。

说话人 1
那你就是我们这个团体，就是进展过程中也，嗯，提到了饮食运动方面嘛。那你就是经过这个团体之后，你在生活中的饮食行为跟运动方面有怎样的新的认识或者变化吗？

说话人 2
嗯，我饮食方面的话，我会，我明显感觉我自己有所减少，就是相对于平时的话，我可能进食会适当的控制减少了。然后运动的话，前期就是运动会，嗯，比较多，但是后期的话感觉自己的身体有一点，嗯，支撑不住了，就是有一点疲劳，较累，然后我就进行了一个舒缓，就是没有那么强烈的运动。

说话人 1
那你现在运动是一个怎么样的状态？现在。

说话人 2
就是想起来。

说话人 1
噢噢，就是没有过度的要求。对，现在。

说话人 2
就是随时就。嗯，有空就去。

说话人 1
那你就是对这些就是你进食行为跟运动的一些改变，你自己有什么样的想法，或者带给了你什么影响？

说话人 2
我觉得运动让我的身体变得更轻松活腻了吧。我其实如果长期运动坚持下来的话，我感觉我自己整个人，嗯，精神状态比较良好，然后身体也没有那么疲倦。就是适量的运动，没有像那种疲倦感了。

说话人 1
那你怎么看待情绪性近视呢？

说话人 2
情绪？我觉得我自己倒是没有经常就是因为什么愤怒、烦恼而过度进食的，这个点就是没有体会过。

说话人 1
嗯，好。那你这个团体结束之后，你之前入组就是入我们团体的期待是否得到了满足？

说话人 2
我最开始进来是想要减肥的，然后我在这个团妇当中我确实也瘦了，然后也没有那么焦虑了，就是减肥方面也收获了更多的知识，其实我觉得还是很有，还对我减肥很有帮助的。

说话人 1
嗯，好。那有没有就是有哪些遗憾或者是期待是没有满足的呢？

说话人 2
遗憾的话就我因为昨天我自己个人原因没有参加到最后的交换礼物环节，其实我是准备了礼物的，一直放在寝室，但是因为自己私人原因没有参加，最后没有圆满结束，其实自己也挺遗憾的。

说话人 1
那在团服过程当中你做了哪些具体的努力来帮助自己实现你的这个减重的目标？

说话人 2
我之前每天都会坚持跳帕梅拉、跑步这些运动。

说话人 1
就是会在运动方面可能就是频率更高了，频率更高。嗯，那在我们这个团服的过程当中，你自身有什么样的变化？就是总的来说的话。

说话人 2
嗯，我觉得我就是因为我们寝室是有一面镜子的，我可以随时观察到自己，我觉得明显的变化就是我的身体的厚度变小了，就是没有之前那么结实了，然后我的心态也发生了变化，因为我之前的话看见我的体重如果不减就会很焦虑，但是实际上它体脂肪它就是一个慢消耗的过程，然后经过团腐活动就是解释这些，然后我现在也没有那么焦虑了。

说话人 1
那你现在就是对你自己的这些变化，你有怎么样的感受呢？

说话人 2
我觉得顺其自然。嗯，具体的话我觉得只要坚持下来就一定会有改变的，就是按着这个方法坚持。

说话人 1
对于你自己现在的一个整体的状态，你有，你是作何评价？你感觉你自己现在是什么样的？

说话人 2
我觉得我还没有减到我想要的体重。嗯，然后我会继续坚持下去的。

说话人 1
就是。嗯，怀春是希望继续，对，努力。嗯，对。那你的这些改变就是你现在一些变化是怎么发生的呢？他是怎么就是他就是你自己觉得在这我们团富过程当中，那么它是怎么发生的？就是就哪些因素促进你有这些改变？你觉得或者有动力？

说话人 2
一个来自身边朋友的鼓励，然后一个就是我会看见比较健康的身材的话，我自己是会有一种羡慕，就是有那种动力去减。

说话人 1
就是外界的推动力。对对对。嗯，那你觉得你现在的一些变化对你生活有什么影响呢？

说话人 2
嗯，明显没有那么的身材焦虑了，然后对自己的尺码也是没有那么多顾虑了，吃东西的话也不会，嗯，更多的就是害怕别人的目光这些在意点呢。

说话人 1
你觉得我们这个团体对你最有帮助的地方是什么？

说话人 2
嗯，我觉得对我心理上的安慰就是因为我之前减肥特别焦虑，经过这次团服我感觉我没有很特别。

说话人 1
那你觉得我们的团体最大的一个特点是什么？

说话人 2
嗯，轻松愉快，就是整体上是比较轻松的，而且也是。嗯，朋友之间交流聊天比较舒服的一个过程。

说话人 1
那你觉得我们这个团服就是有没有哪些方面做得不够好的？就是可以改善的地方？

说话人 2
嗯，我没有觉得什么不够好，但是如果提意见的话，我觉得以后可以。嗯，做一些小游戏，就是那种团体小游戏，可以更加的尊敬友谊，就是互动这些。然后一些就是那种同时也能锻炼运动量，就是一些小小游戏。

说话人 1
那如果要你给你身边有类似的就是减重需求的同学推荐我们这个团服，你会怎么说呢？会怎么推荐？我说。

说话人 2
你愿意跟我一起去吗？哈哈哈，我，我有一个减肥的团服，如果你有减肥的需求的话，可以愿意跟我一起去。

说话人 1
就是告诉他我们这个团服是可以达到减肥效果的，是吧？对，嗯，好，那么就差不多这些。好，谢谢你。

说话人 2
谢谢，现在不是要聊那个吗？嗯，一会聊。

说话人 1
晚上聊吧。我什么时候给你发消息嘛？那你看一下，我看见了你回复我一下，我就过去。
