# Supplementary material for: Helpful factors of group cognitive behavioral therapy in overweight and obese college students
Source: Front Psychol. 2025 Sep 12;16:1585765. doi: 10.3389/fpsyg.2025.1585765 (PMC12463828; doi:10.3389/fpsyg.2025.1585765)
Supplement: Supplementary file 11 [file Supplementary_file_11.docx]

**伯程 1889**

*2024年7月17日 下午 10:54
9分钟 15秒*

**关键词**

团体 分享 焦虑 饮食 收获 知识 痛苦 经验 运动知识

**文字记录**

说话人 1
那我们现在开始吧。

说话人 1
嗯，请分享一下你在我们这个团体中的一个整体的一个感受和体验。

说话人 2
整体感受还是比较愉悦欢快的吧。

说话人 1
因为我们团体也经过了 8 次，那你随着这个时间的一个推移，你的感受它有经历怎样的变化？

说话人 2
嗯，就是在最开始开前面几次那个团体的时候，还是就是有一点不是特别信赖的感觉。然后到后面就慢慢的发现了这个团体带来的一些收获，还有他的一些魅力，然后就也能更全线性的投入到这个团体中。

说话人 1
嗯，就更相信我们团体了，是吧？好，那你在我们这个团体当中有哪些事件是给他留下了深刻的印象，或者是哪些时刻？

说话人 2
嗯，我觉得就是在后面几次，每次的那个分享总结的那个地方，就是能听到大家的一些反馈，还有分享的一些经验和方法都特别深刻那些。

说话人 1
但你当时的给一个感受是什么呢？

说话人 2
当时感受就是，嗯，那种茅塞顿开的感觉，就是感觉知道了很多新奇的东西。

说话人 1
那，嗯，这些事情就是你印象深刻的这些事情对你有什么影响呢？

说话人 2
印象深刻的就是在我后面的，在后面的减肥过程中就是会联想到是嗯团体中的那些内容，经验就会去思考，然后也会把它们运用在后面的减肥计划中。

说话人 1
那因为我们团妇也经历了 8 次，那在这个过程当中，嗯，就是他对你的日常生活的运动方面，或者是进食行为方面有怎样的变化吗？

说话人 2
嗯，在运动方面的话就是知道了那个运动还是不能特别单一的只进行一项运动，就是可以多种运动，互相的协调，然后这样进行锻炼，然后在饮食方面就是控制自己吃饭的那个量和吃饭的速度。还有就是一个正面饮食，然后平常食物的那个味道，然后控制那个吃饭速度嘛？

说话人 1
那就是刚刚运动，你说了就是你可能运动知识上可能有更多的体会，那你平时运动的频率这方面没有什么。

说话人 2
影响，平时运动的频率我觉得到后面的话，就是对于运动的话就是更上进了，就是更愿意去做。在以前感觉运动它还是有一点强迫性的。嗯，只是在那个过程还是有一点痛苦的。嗯，但是到后面那种痛苦就慢慢的减弱了，更多的就是一种享受。

说话人 1
嗯，那你在运动和饮食方面的变化，它对你有什么影响吗？

说话人 2
嗯，在体重上还是能感觉到，就是掉了一些，然后我觉得运动了感觉更自信一点了。

说话人 1
你有没有情绪性进食的行为？

说话人 2
情绪性进食没有，吃饭都比较，就是比较健康的，然后吃的也不算准时，因为有时候总是不吃。但是吃饭的时候都是在那个点。

说话人 1
嗯嗯，那你就是你现在团府结束了，那你曾经就是进入我们团府的钱的一些期待得到了满足。

说话人 2
嗯，还是大部分都满足了，我毕竟在那个知识上也有所收获，然后体重上也有明显的变化。

说话人 1
嗯，那我在我们这个团腐的过程中，你自己付出了哪些努力来帮助自己实现这个减重目标？

说话人 2
嗯，就是根据老师说的那些方法，然后自己控制吃饭的量，就是把改掉一个健，改成一个健康的饮食习惯，然后运动的话也是有在经常的坚持。嗯，所以就还是有一点改变的。

说话人 1
那你，嗯在我们这个团富过程中，你自身整体有什么样的变化？

说话人 2
自身整体上，整体上感觉还是有变得那么自信一点了。

说话人 1
那你怎么如何评价你现在的一个状态呢？

说话人 2
我去，现在状态还是特别积极乐观的。嗯，就是愿意去做更多的事。

说话人 1
那平常就焦虑情绪这些有变化吗？

说话人 2
嗯，在团普之前的话确实经常就是焦虑这些方面。然后大家好，在团舞之前就是经常容易焦虑这些方面，然后在后面的话现在就好多了，就是没那么没想那么多东西了，特别就是在体型焦虑这方面。

说话人 1
好，你电话急吗？要保时捷。没事，那我们继续吧。嗯，那你觉得是哪些因素促成了你现在的一个变化和成长？

说话人 2
嗯，就是在达到减重目的过后的那些变得更好的感觉，就是无论是在他人在面对别人，还是自己在做一些事儿的时候。

说话人 1
嗯，就是你自己本身带的一种期待跟他的一些鼓励。那你觉得我们这个团服最有帮助的地方是什么？

说话人 2
最有帮助的地方就是找到了一群志同道合的人，然后我互相的分享，互相的监督，然后鼓励一起达到一个目的。

说话人 1
那你在这个团体当中有没有哪些遗憾？是，就是没有完成的。

说话人 2
遗憾。我觉得倒没什么吧？挺好的。

说话人 1
嗯，你觉得我们这个团服最大的特点是什么？

说话人 2
最大的特点，嗯，就是特别的包容。然后，对，就是有的时候，对那些同学他们没有及时来，或者有事，然后请假的那些，就是也不会过多的追究什么，然后就是考虑的也特别周到，就是在那个送礼物的时候也是考虑了那些没送的人让他们请人带。

说话人 1
那你觉得我们团妇有没有哪些地方做得。

说话人 2
不好的地方？我觉得就是每次，虽然它是一个偏理论的那种，但是每次在那个地方坐一个半小时还是有点累。

说话人 1
就是可能需要一些更加活跃的方式。对。

说话人 2
就是可以在那个中间稍微的穿插一些活动之类的。

说话人 1
那如果你要给你周围有类似的减重需求的同学推荐我们这个团妇，你会怎么推荐呢？

说话人 2
嗯，就是把我在团服中的那些收获就是分享给他们。

说话人 1
嗯，就是告诉他你在这里有得到就是达到一些目标，是吧？嗯，好，那基本上就这样，好，好，谢谢。嗯，好的。
